# Designing strategic scenarios for the digital transition

**DOI:** 10.1038/s41598-026-52549-w

**Published:** 2026-05-14

**Authors:** Vítor Alcácer, Alexandra Tenera, Francisco Araújo, Helena Carvalho, Virgilio Cruz-Machado

**Affiliations:** 1 Instituto Politécnico de Setubal, Escola Superior de Tecnologia de Setubal, Setubal, 2910-761 Portugal; 2 DICE Lab, Escola Superior de Tecnologia de Setubal, Instituto Politécnico de Setubal, Setubal, 2910-761 Portugal; 3 ALGORITMI Centre, Universidade do Minho, Guimarães, 4800-058 Portugal; 4 Department of Mechanical and Industrial Engineering, Faculty of Science and Technology, Universidade Nova de Lisboa, Caparica, 2829-516 Portugal; 5 UNIDEMI, Research and Development Unit for Mechanical and Industrial Engineering, Faculty of Science and Technology, Universidade Nova de Lisboa, Campus da FCT/UNL, Caparica, 2829-516 Portugal; 6 Laboratório Associado de Sistemas Inteligentes, LASI, Guimarães, 4800-058 Portugal

**Keywords:** Industry 4.0, Strategic scenario development, Risk assessment, Risk management, Business and management, Business and management, Operational research

## Abstract

Strategic scenarios support decision-making under uncertainty; however, existing Industry 4.0 (I4.0) research predominantly adopts technological perspectives and does not provide methodologies that integrate risk management into scenario development. This study addresses this gap by proposing a methodology for developing strategic scenarios for I4.0 adoption based on risk assessment and risk appetite. Following a Design Science Research approach, the study includes a systematic literature review (20 studies) to examine existing scenario development approaches in I4.0, which did not identify any methodologies incorporating risk management. This is complemented by a qualitative case study involving 15 experts from industrial and academic contexts. A structured risk analysis identified 35 risks across 9 categories, enabling the prioritization of key factors influencing digital transformation. The study results in the development of a validated methodological framework and four strategic scenarios derived from the interaction between risk levels and organizational risk appetite. The findings demonstrate how integrating risk management into scenario planning enhances strategic decision-making under uncertainty. This study contributes by addressing a critical gap in risk-based scenario methodologies for I4.0 and by proposing a novel framework that integrates risk management into strategic scenario development.

## Introduction

 Industry 4.0 (I4.0) represents a paradigm shift in industrial systems, characterized by the integration of digital and physical processes through cyber-physical systems, data analytics, and interconnected technologies. These developments enable higher levels of automation, flexibility, and efficiency, allowing companies to optimize production processes and respond more dynamically to market demands^1,2^. However, the benefits of I4.0 adoption are not uniformly distributed, as they depend on organizational capabilities, resource availability, and the ability to manage technological complexity and uncertainty.

This challenge is particularly critical for Small and Medium-sized Enterprises (SMEs), which constitute most industrial actors and often face limitations in financial resources, technological infrastructure, and specialized knowledge^3^. In such contexts, the adoption of I4.0 is not merely a technological decision but a strategic one, involving multiple stakeholders and requiring alignment between organizational capabilities and external environmental conditions^4^. Previous studies have identified key barriers to I4.0 adoption, including the lack of clarity regarding economic benefits, insufficient interoperability standards, and underdeveloped IT infrastructures^3,5^. These barriers increase the complexity of decision-making processes and reinforce the need for structured strategic approaches.

Despite these challenges, many organizations, particularly SMEs, continue to approach I4.0 adoption without systematically integrating risk into strategic decision-making. This limitation often leads to inefficient investments, particularly in technologies with uncertain returns, and increases exposure to technological obsolescence as innovation cycles accelerate^6,7^. The absence of structured, risk-informed planning frameworks makes it difficult for companies to anticipate uncertainties, evaluate trade-offs, and align strategic decisions with their risk tolerance. According to ISO 31000:2018, risk is defined as the effect of uncertainty on objectives, highlighting the importance of integrating risk management into strategic processes. However, this integration remains largely absent in existing approaches to I4.0 planning and scenario development^8^.

Recent developments in digital transformation further increase the need for more sophisticated strategic approaches. Data-driven design, advanced analytics, and digital manufacturing systems are enabling highly customized, simulation-based, and interconnected production environments, fundamentally reshaping industrial value creation^9,10^. For instance, recent studies such as Lu et al.^11^ integrating machine learning, finite element analysis, and optimization techniques demonstrate how digital technologies enable highly customized, simulation-based, and data-driven product development processes. These developments expand the scope of strategic decision-making by introducing new interdependencies, accelerating innovation cycles, and increasing exposure to uncertainty. As a result, organizations must move beyond purely technological perspectives and adopt strategic frameworks capable of addressing both opportunities and risks associated with digital transformation.

Strategic scenarios have been widely recognized as valuable tools for supporting decision-making under uncertainty, as they allow organizations to explore alternative future developments and assess the implications of different strategic choices^12,13^. In the context of I4.0, scenario-based approaches can help organizations anticipate technological evolution, identify potential challenges, and design more resilient strategies.

Although prior studies have explored I4.0 scenarios and strategic planning approaches, existing contributions are predominantly focused on technological or operational perspectives, lacking the integration of risk management into scenario development^14^. This limitation reduces the ability of organizations to make robust strategic decisions under uncertainty. Accordingly, this study proposes a methodology for developing strategic scenarios for I4.0 adoption grounded in risk assessment and risk appetite.

Unlike existing approaches, the proposed framework explicitly integrates risk management into scenario development, enabling organizations to systematically incorporate uncertainty into strategic decision-making. Following a Design Science Research (DSR) approach, the study develops and validates a structured framework through a combination of systematic gap identification and an empirical case study. In doing so, this study contributes to both theory and practice by advancing risk-informed scenario planning and providing organizations with a practical tool to support decision-making in complex I4.0 environments.

Furthermore, the literature on scenario development has been characterized by a lack of methodological consensus, often described as a “methodological chaos,” reflecting the diversity of approaches and the absence of standardized frameworks^15,16^. This fragmentation becomes even more problematic in the context of I4.0, where the complexity of technological and organizational transformations requires more structured and integrated approaches. There is a lack of methodologies that explicitly incorporate risk assessment and risk appetite into the development of strategic scenarios, limiting the ability of organizations to make informed decisions under uncertainty.

To address this gap, this study proposes a methodology for developing strategic scenarios for I4.0 adoption grounded in risk assessment and risk appetite. Following a Design Science Research approach, the study aims to develop and validate a structured framework that supports organizations in integrating risk management into scenario planning processes. Based on this objective, the following Research Questions (RQs) are formulated:

RQ1) What are the methodologies to develop strategic scenarios for Industry 4.0 adoption based on its risks?

RQ2) What are the strategic scenarios for Industry 4.0 adoption based on its risks?

RQ3) What are the main risks associated with Industry 4.0 adoption?

By addressing these questions, this research contributes to advancing the literature on scenario development and provides practical guidance for organizations seeking to navigate the complexities of digital transformation. The remainder of this paper is structured as follows: Sect. 2 explores current scenario development and its variables, Sect. 3 shows the methodology used in this research, Sect. 4 presents the proposed methodology to develop strategic scenarios, Sect. 5 presents a case study application of the proposed methodology, Sect. 6 discusses the obtained results and Sect. 7 ends this research with the conclusions, research limitations, and further research studies developments.

## Theoretical background

The unpredictability of technological advances requires high flexibility and adaptability on the part of companies and public institutions. Anticipating future developments and their implications is essential for effective planning and execution^17^. The sooner companies and public institutions identify opportunities and develop solutions, the greater the success achieved^18^.

There is still no consensus on the definition of a scenario. For Foster^19^ and Schoemaker^12^, a scenario is a future image developed through the interconnection of quantitative and qualitative elements. A different viewpoint is mentioned by Ratcliffe^20^, that describes scenarios based on the following elements: (i) reports describing different future projections; (ii) instruments that support the improvement of quality and robustness in strategic decisions; (iii) mechanisms that evaluate the effectiveness of current strategies considering future objectives. Van der Heijden^13^ defined scenarios as instruments that allow the identification of the development of the driving forces, and their interdependencies, establishing connections between these forces, emerging opportunities, and associated risks.

Taking into account the evolution of external factors, Börjeson et al.^21^ defined strategic scenarios as descriptions of the possible consequences of internal decisions controlled by agents. These exploratory scenarios answer the question “what might happen?”, presenting different perspectives and possibilities to analyze the impacts of alternative events.

The divergence of author’s opinions is based on their definitions on different types of scenarios. The literature continues to reveal some difficulty in reaching consensus on the definition of what constitutes a scenario^22^. Spaniol and Rowland^15^ highlighted that the lack of consensus becomes even more evident when scenario development is characterized as a real “methodological chaos”, evidenced, aligned with Bradfield et al.^16^, by the multiplicity of often contradictory definitions, as well as by the diversity of associated characteristics and methodologies. In this study, the strategic scenario definition adopted is the following: strategic scenarios are projections of events that can occur in the future, being consequences of strategic decisions in the present.

Strategic scenarios enable the identification of a set of possible consequences of strategic decisions, offering relevant insights to decision-makers and stakeholders. These, as interested parties, include all those affected by an organization’s decisions. Strategic scenarios, with both a qualitative and quantitative approach and generally focused on the long term, focus on internal decisions under the control of agents, without disregarding the influence of external factors^21^.

The development of scenarios for emerging technologies implies a high degree of uncertainty, which compromises the reliability of traditional forecasting methods^17,23^. Thus, the value lies in creating alternative scenarios with different perspectives, not in exact prediction, as this allows companies to explore possible technological directions, promoting competitiveness and facing uncertainty^18^. Investment in I4.0 should follow a continuous improvement approach and companies should consider feasibility issues related to data preparation and infrastructure to allow decision-makers to cost-effective decision-making. In companies, the influence of the stakeholders is crucial for I4.0 adoption^24^. Thus, the development of the decision-making process involves the convergence of the involved people, and the decision-making evaluates scenarios considering different alternatives, following the best option, and performing the needed actions^25^. Related to decision-making, strategic management plays an important role in modern companies. Managers, leaders, and business owners empower long-term goals, focus, and plans to face companies’ major challenges. They define how and where the companies’ position, how it can be developed and deploy valuable resources to be competitive and to achieve companies’ performance targets^26^.

Among other aspects, strategy deals with risk approach, risk culture, risk attitude, and risk appetite. To develop and reach a strategy, managers must implement risk management in their companies^27^. Risks can be categorized as financial, hazard, operational and strategic risks. The strategic risks are the category of risks that can be assumed as the companies’ obstacles to achieving their strategic goals. Also, the strategic risks can decrease companies’ status, customer satisfaction and technological innovation^28^.

In this research, the “risk” definition adopted is “the effect of uncertainty on objectives” as proposed by the ISO 31000:2018 standard^8^, which can produce positive and negative impacts, as opportunities and threats. In the business context, risks can be classified as internal (linked to organizational factors, capacity, or information failures) and external (price changes, competition, production costs, supplier quality, or political factors). Identifying risks is essential, as the precision of the scenarios is directly linked to the accuracy and reliability of the collected information^29^. In the literature, the terms “risk” and “risk factor” are used by some authors without a clear distinction. However, Moeuf et al.^30^ identified main challenges, risks and the factors that impact the success, risks associated with the I4.0 implementation. Birkel et al.^6^ presented a comprehensive list of risks associated with the I4.0 context. Sanchez^31^ conducted an exploratory analysis of the main risks and challenges associated with I4.0 implementation. Furthermore, it was highlighted the difficulty companies have in the fit of new methods, and the slowness of some of them in I4.0 adoption. A system of risk management adjusted to the emerging challenges of I4.0 adoption was presented by Tupa et al.^7^, highlighting two particularly significant risks.

Macurová et al.^32^ identified risks, main barriers, and the driving forces of I4.0 based on literature and based on industrial companies in the Czech Republic. Niesen et al.^33^ research aimed to develop a risk management framework focused on I4.0 process data. According to the literature review, a proposed list of Risks (R) and associated risk categories (RC) to the I4.0 process is shown in Table 1.

To ensure a structured and comprehensive identification of risks associated with I4.0 adoption, this study categorizes risks into nine key dimensions. These categories were derived from the literature by grouping recurrent risk factors into coherent domains that reflect both technological and organizational challenges. Specifically, the selected categories encompass critical areas such as cybersecurity, data management, infrastructure, workforce capabilities, and strategic alignment, which are consistently identified as major sources of uncertainty in digital transformation contexts. This categorization aims to provide a holistic view of the risk landscape, ensuring that both technical and non-technical dimensions are considered. By structuring risks into these nine categories, the study establishes a foundation for systematically analyzing their impact and supporting the development of risk-informed strategic scenarios.

**Table 1:** Risks and risk categories.

Risk categories	Risks
RC1 - Cybersecurity	R1 - Loss of confidential information^6,7,31,33^ R2 - Espionage in industry^33^ R3 - Sabotage in industry^31,33^
RC2 - Labor	R4 - Disappearance of jobs^6,31^ R5 - Lack of qualified labor^6,7,30–32^ R6 - Training with new requirements^6^ R7 - Perception of increased vigilance due to I4.0^30^
RC3 - Competition	R8 - Emergence of new competitors^6,32^
RC4 - Organizational structure	R9 - Lack of strategy in the long term^30^ R10 - Organizational culture and resistance^6,31^ R11 - I4.0 implementation difficulties^31^
RC5 - Infrastructure	R12 - Reliability of the infrastructure^6,33^ R13 - Maturity of the technology^6^ R14 - Use of network infrastructures^6^ R15 - Interoperability^6^
RC6 - Legal	R16 - Protection of data^6^ R17 - Increasing legal complexity^6^
RC7 - Sustainability	R18 - Waste^6^
RC8 - New business models and market conditions	R19 - Instability of demand^32^ R20 - More demanding requirements for product individualization^32^ R21 - Knowledge of a data-driven business model^6^
RC9 – Financial	R22 - Technological obsolescence^30,31^ R23 - Inefficient investments^6^ R24 - Cost of labor^6,31^ R25 - Uncertain return on the investment^6,31^

Recent studies have increasingly emphasized the strategic and organizational dimensions of digital transformation in manufacturing contexts, particularly for SMEs. For instance, Kavre et al.^34^ identify key digitization strategies such as the development of strategic roadmaps, effective change management, and workforce training, as critical enablers of digital technology adoption in manufacturing Micro and SMEs. Similarly, Kavre et al.^35^ highlight how barriers to digital disruption in cleaner cloud manufacturing environments are not only technological but also organizational and strategic in nature, including limitations related to skills, infrastructure, and managerial readiness. These contributions reinforce that I4.0 adoption extends beyond technological implementation, requiring structured strategic approaches that account for organizational capabilities and systemic constraints.

Despite the growing body of literature on I4.0, digital transformation, and scenario development, existing studies predominantly focus on technological, operational, or descriptive perspectives, including more recent strategy and sustainability-oriented approaches. Limited attention has been given to the integration of risk management into the development of strategic scenarios, particularly in the context of I4.0 adoption. Moreover, while previous research identifies various risks and barriers associated with digital transformation, these factors are rarely systematically structured and incorporated into scenario-based strategic decision-making frameworks. This gap highlights the need for methodologies that explicitly integrate risk assessment and risk appetite into the development of strategic scenarios, providing organizations with more robust tools to navigate uncertainty. Addressing this gap constitutes the main objective of this research.

## Research method

In this study, surveys and methods were carried out per relevant guidelines and regulations. The NOVA School of Science and Technology of NOVA Lisbon University’s institutional and licensing committee approved all experimental protocols. Informed consent was obtained from all subjects and legal guardian(s).

**Fig. 1:**
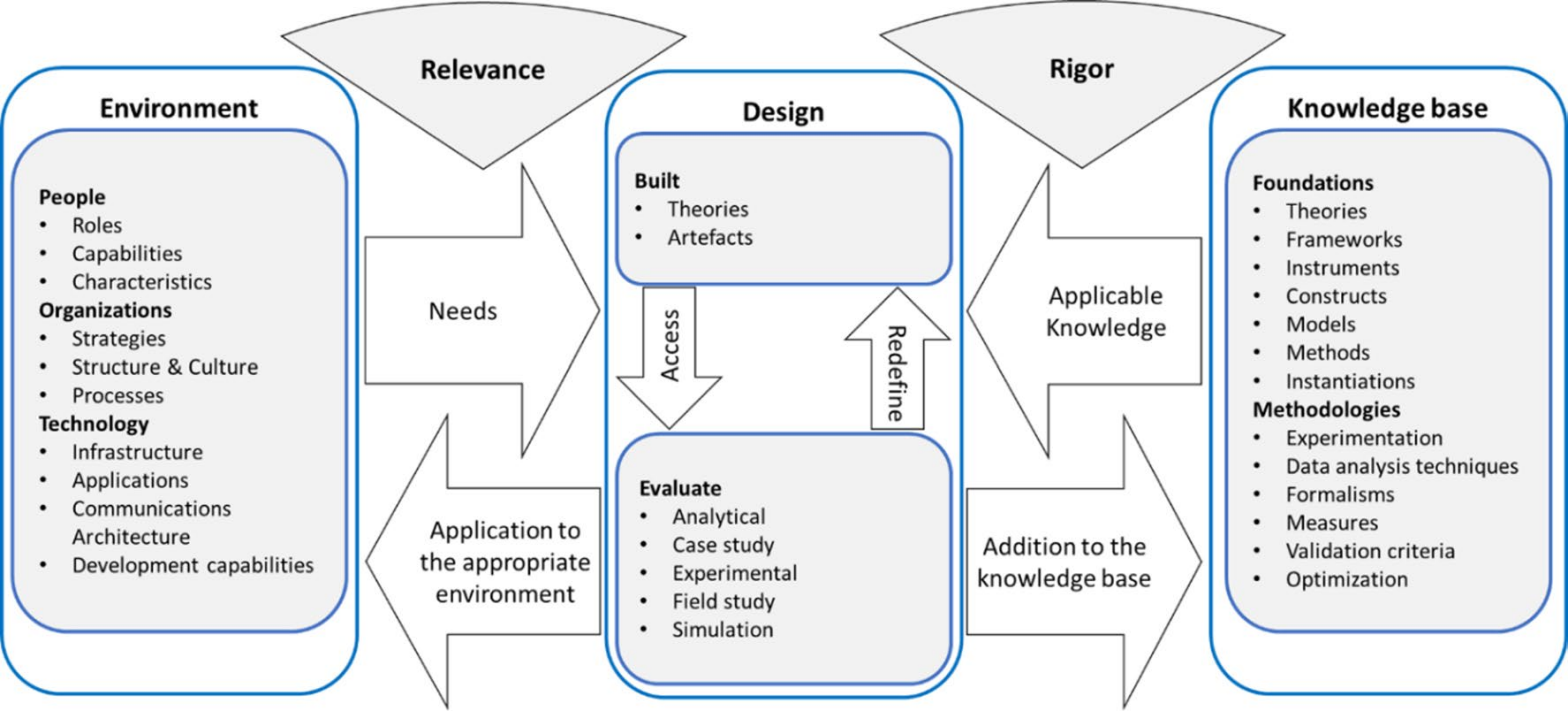
Conceptual framework of DSR. **(adapted from vom Brocke** et al.^38^.**)**

**Fig. 2:**
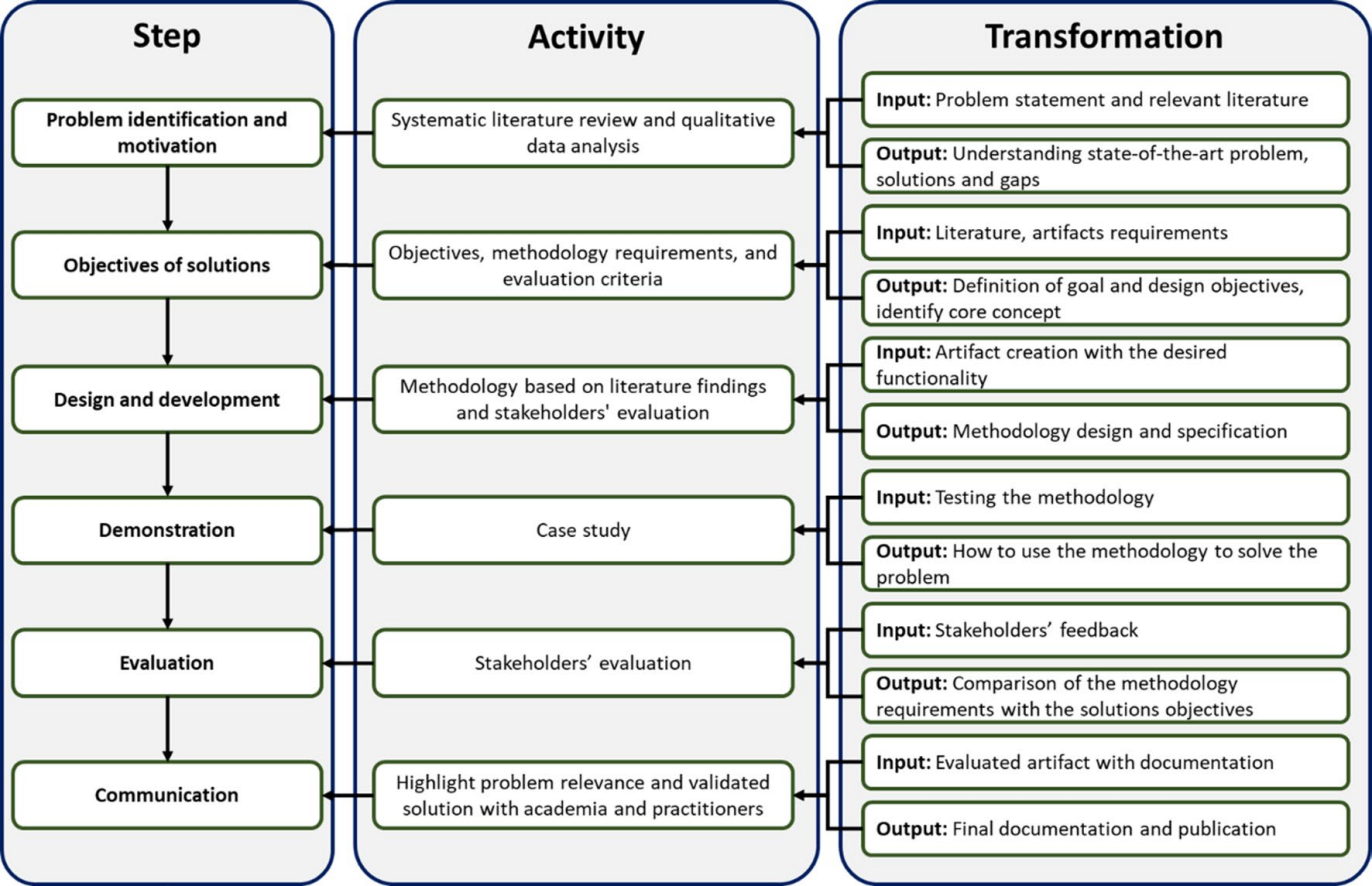
DSR methodology applied for this research^37^.

The research approach adopted in this study aimed to identify methodologies for developing risk-based strategic scenarios and to characterize the resulting scenarios of I4.0 adoption. Thus, to structure the development of theoretically grounded instrumental knowledge, the Design Science Research (DSR) method is a valuable framework to ensure academic and practical relevance. DSR aims to provide innovative solutions to real-world problems while advancing knowledge through the development of artifacts, focusing on problem-solving artifacts development with a clear contribution to the body of knowledge^36^. Figure 1 shows the framework of the DSR conceptual framework, and Fig. 2 shows the adopted process model in this research to implement the DSR with the six steps of Peffers et al.^37^.

DSR methodology arises from a relevant problem from the environment, following an abductive phase, i.e., it is intended to solve the problem emerging from the environment to create a novel element taking advantage of the existing knowledge, leading to the generation of new knowledge as a form of artifact, such as a methodology. Next phase is deductive to validate the proposed artifact through tests^36^.

The case study conducted in this research was based on a sample of stakeholders and companies from the Setubal peninsula in Portugal. AISET (an industrial organization in the Setubal peninsula) and two higher school institutions from the Setubal peninsula also participated in this sample. Therefore, companies in the Setubal Peninsula are suitable for developing and testing the artifact. The close collaboration with Setubal peninsula companies allowed the development of an understanding of the problem and the analysis of the artifact.

The data collected for this study were primary data from literature review, the stakeholders’ panel, and the specialists’ panel. Interviews were conducted with stakeholders and specialists, and several brainstorming sessions with the researchers to validate data collected. The validation of the methodology was conducted through researcher brainstorming sessions and with the experts’ panel during the testing of the methodology.

## Proposed methodology to develop strategic scenarios

By answering the question “What might happen if we act like this?”, strategic scenarios explore the possible consequences of strategic decisions, considering different future developments. They are particularly useful for supporting decisions and guiding the vision of stakeholders. The main purpose is to act as an integrated tool that facilitates understanding of the trajectory of technological advances, reducing uncertainty and increasing the chances of achieving established objectives.

**Fig. 3:**
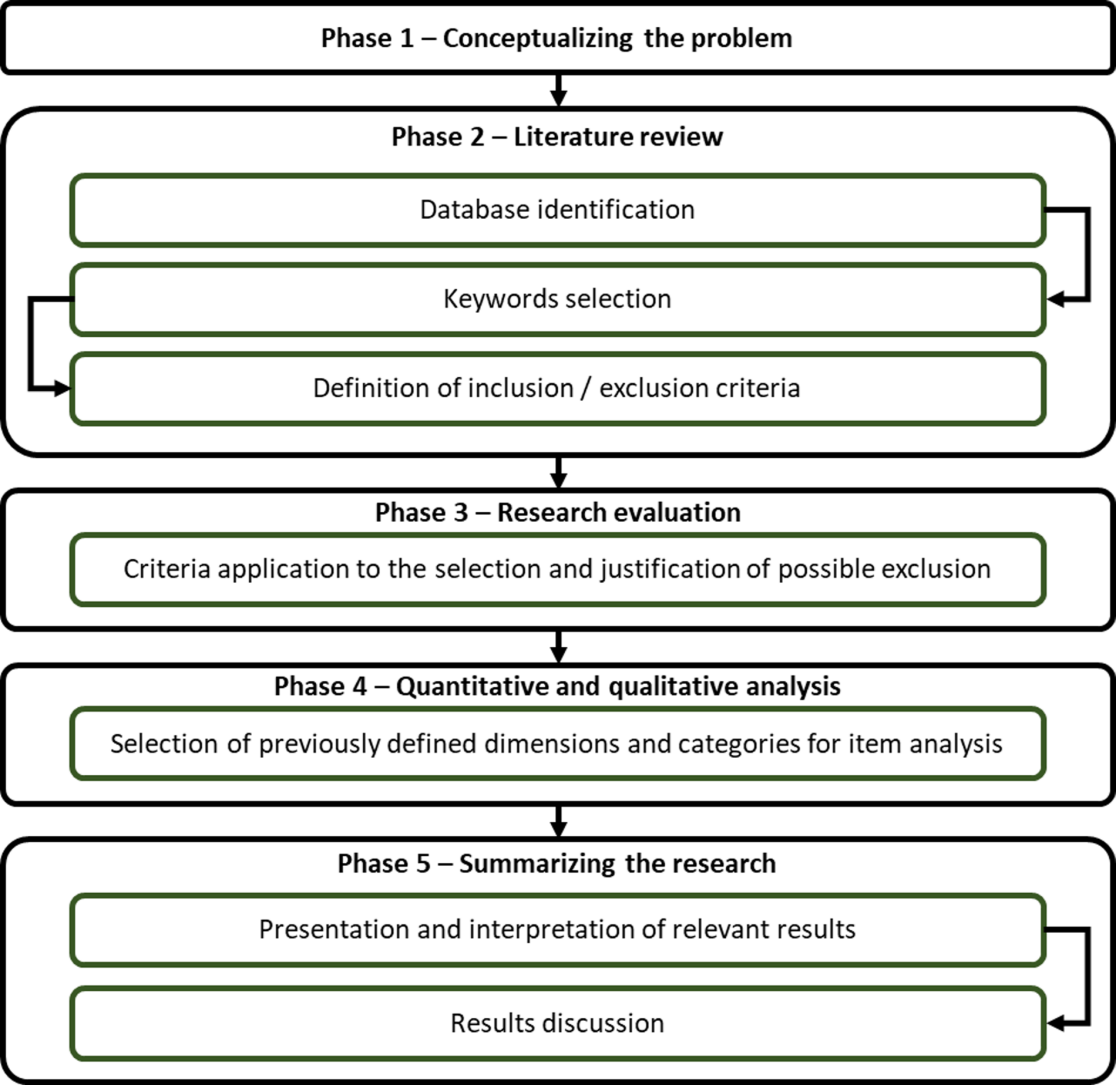
SLR approach. **(adapted from Espadinha-Cruz** et al.^39^.**)**

A SLR (Systematic Literature Review) was conducted to identify methodologies to develop strategic scenarios for I4.0 adoption based on risk analysis and management, following the adapted five steps in Fig. 3of Espadinha-Cruz et al^39^. A SLR is objective, reproducible, and less prone to bias regarding the research authors^40,41^.

To ensure the quality of the sample, the scientific databases SCOPUS and Web of Science (WoS) were used for the collection of articles, and only peer-reviewed and indexed publications were considered^42^. Only journal articles, journal reviews, conference papers, and book chapters written in English were considered. The search was conducted without temporal restriction to capture the full scope of existing research on scenario development in I4.0. Additionally, studies that did not address strategic or scenario-based approaches were excluded from the analysis to ensure alignment with the research objective.

To obtain a relevant answer to the RQ1 in a larger number of studies, the keywords “scenario methodology”, “strategic scenario development”, “scenario development” “predicting future scenarios”, “predicting scenarios”, “future scenario development”, “future scenario*” were combined with “industry 4.0”, without limitation of publication year as well. The search strategy included identifying studies containing the selected keywords in the title, abstract, or keywords in the study. This search resulted in 31 studies in SCOPUS and 17 studies in WoS, i.e., a total of 48 studies were found, with 13 common database studies. Inclusion criteria were defined to eliminate studies that did not serve the purpose of the study. By this, the title, abstract, keywords, and context of each study were analyzed and only studies matching the selected keywords were considered. After applying inclusion criteria and excluding studies that do not match the purpose, 20 studies were considered for the final analysis, 6 studies from the SCOPUS database, 1 study from the WoS database, and 13 studies were common to both databases.

A content analysis was performed to analyze the results of the SLR. Table A.1 in the appendix A shows the 20 studies analyzed and it can be concluded to answer the RQ1 that no article proposes a methodology to develop strategic scenarios for the adoption of I4.0, based on its risks. None of the identified studies explicitly combined risk assessment and risk appetite within scenario development frameworks. Thus, it is necessary to propose a methodology in this matter to fill this gap.

**Fig. 4:**
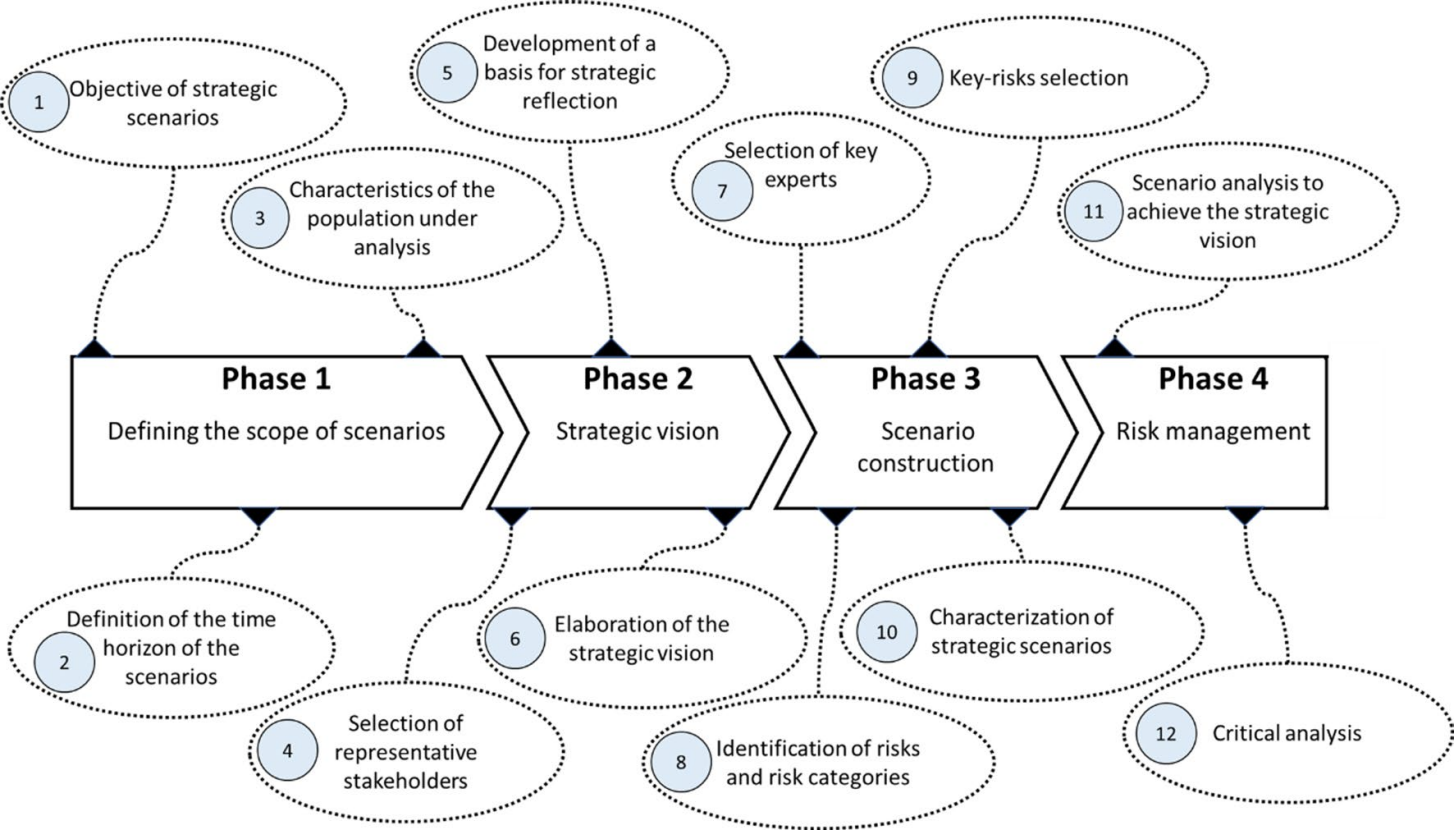
Proposed methodology to develop strategic scenarios.

The construction of a methodology for developing strategic scenarios based on the integration of risk assessment and risk appetite into scenario development, as assumed in this study, in Fig. 4, started with the approach given by Fotr et al^22^, which identifies three typical phases in the scenario formation process, namely, choosing participants to be involved in the scenario formation process (step 4 and step 7), determination of driving forces and futures events (risks or uncertainties) (risks assumed in steps 8 and 9), and scenario elaboration process (step 10) and consistency testing (step 11 and 12). Additionally, to include risk management in the development of strategic scenarios, in this methodology’s development, it was added the steps of the risk management process of ISO3100:2018 standard ISO^8^, namely, the scope, context, and criteria phase (step 8), risk assessment phase (risk identification (step 8), risk analysis (step 8), and risk evaluation (step 9)), and risk treatment phase (step 12). This methodology also contains a strategy vision phase to serve as a comparative analysis of the strategic scenarios in step 11. The proposed methodology in Fig. 4 was evaluated through a brainstorming session with the stakeholders’ panel of the case study presented in Sect. 5. During these brainstorming sessions, feedback from stakeholders and experts led to iterative refinements of the proposed methodology. For example, expert feedback led to the reclassification of certain risks based on expert consensus, and to adjustments in the definition of strategic scenarios, particularly regarding the distinction between high-impact and high-uncertainty risks. Discussions contributed to the adjustment of risk categorization, the clarification of key assumptions underlying the strategic scenarios, and the refinement of the strategic vision for the defined time horizon. These interactions ensured that the methodology evolved through continuous validation and alignment with practical industry perspectives, reinforcing the abductive nature of the DSR process.

### Defining the scope of scenarios (Phase 1)

Strategic scenario development begins with defining its scope, and it is essential to clarify the purpose and objectives to encourage its creation^22,43^. This phase identifies the specific characteristics of the strategic scenarios that are of stakeholders’ interest, such as the time frame of the scenario development effort.

#### Objective of strategic scenarios (Step 1)

Establishing a meaningful purpose involves considering issues and outcomes that promote future value for companies^12^. It is essential to identify the main trends and uncertainties that may influence the sustainability of a region or business sector. The main objective of strategic scenarios is to identify the risks associated with the transition to I4.0, highlighting how these threats can impact business models and operations, in addition to the potential consequences of the strategic decisions adopted.

#### Definition of the time horizon of the scenarios (Step 2)

It is essential to analyze the level of predictability and the information available related to future events which can shape scenarios. As the time horizon expands, it becomes more difficult to anticipate factors or events that are still uncertain in the present^44^.

#### Characteristics of the population under analysis (Step 3)

Population must be representative through the analyzed sample so that the results can be generalized. Factors such as population size, demographic characteristics, data availability, number of companies, and their activities directly influence the results of the study.

### Strategic vision (Phase 2)

This phase is essential to develop a strategic vision for the established period. This process is developed in three stages: (1) creation of a basis for strategic reflection to guide dialogue with stakeholders; (2) collection of relevant information; and (3) elaboration of the strategic vision based on content analysis.

#### Selection of representative stakeholders (Step 4)

Representative stakeholders will have the role of collaborating in the joint construction of a vision on the state of development of the population under study. Their selection should be based on criteria that favor experts with experience in leadership and strategic management. This step is fundamental because the success of developing strategic scenarios largely depends on the quality and relevance of the contributions provided by the selected panel.

#### Development of a basis for strategic reflection (Step 5)

It is essential to build a strategic vision base that supports the planning and execution of strategies. This base guide decision-making and the implementation of actions, providing a framework for the study under analysis and promoting an objective discussion among stakeholders. In addition, it facilitates the collection of data on their strategic visions, helping to define the time horizon.

#### Elaboration of the strategic vision (Step 6)

To develop a strategic vision, it may be more appropriate to use qualitative elements. This is because the qualitative approach gives participants greater freedom to share their experiences and build visions, without the limitations of more rigid quantitative techniques. However, because non-numerical elements can be interpreted in many ways and involve poorly defined concepts, it is essential to explore and clarify objectives and key concepts with participants. To support this qualitative data collection, a strategic vision can be developed^45^.

### Scenario construction (Phase 3)

This phase involves the collection, quantification, and validation of the essential contents of the process, focusing on the scenario formulation. All these steps are essential to ensure the robustness of the scenarios developed. Moreover, a panel of key experts is needed for risk identification, assessment, quantification, and qualification. The risks and risk categories are presented to the panel to perform the distribution of the risks according to their level and risk appetite index.

#### Selection of key experts (Step 7)

To make this selection, criteria are developed to include experts whose validations, suggestions, and evaluations are representative of the impacts of these risks on the sustainability of companies. Considering this need, criteria were defined to ensure the selection of experts with relevant experience and knowledge aligned with the study objectives.

#### Identification of risks and risk categories (Step 8)

This stage aims to identify the main risks and respective categories faced by companies in the I4.0 transition. This is a crucial process for construction of the strategic basis, as the effectiveness of the scenarios is related and depends directly on the collected information quality. Accurate and relevant data are essential for developing robust and realistic scenarios^29^. The identification and validation of risks, as well as their categories, can be carried out in two distinct stages:

• A literature review can be conducted to identify and categorize the risks associated with the transition to I4.0;

• Risks and risk categories can be identified and classified in a SWOT analysis to understand whether they positively or negatively affect this transition. In a SWOT analysis, it is recommended to conduct a brainstorming session with a panel of key experts to determine the influence of the identified risks (whether external, internal, positive, or negative) on companies during the transition to I4.0. This analysis is one of the strategic tools suggested to assist in identifying risks^46^.

The next activity is to evaluate each identified risk with the expert panel, considering three parameters: probability of occurrence, potential impact, and risk appetite (refers to the degree of risk that the organization is willing to accept to achieve its objectives before taking corrective measures)^47^. Risk appetite is considered because it reveals a company’s perception and attitude towards risks. A higher appetite can lead to the acceptance of risk without immediate action if the company can manage it. To assess the level of occurrence, impact, and risk appetite, a 6-point Likert scale was developed, ranging from 1 (Very low) to 6 (Very high). This choice considered the size of the scale, the absence of a neutral point, and the meaning of each level, unlike the 5-point scale, which allows neutral responses and can lead to indecision among respondents^48^. Thus, the 4 and 6-point scales were considered. Given that the participants are experts with extensive experience in the area, the 6-point Likert scale was chosen, as it offers greater precision and detail in the responses.

#### Key risks selection (Step 9)

**Fig. 5:**
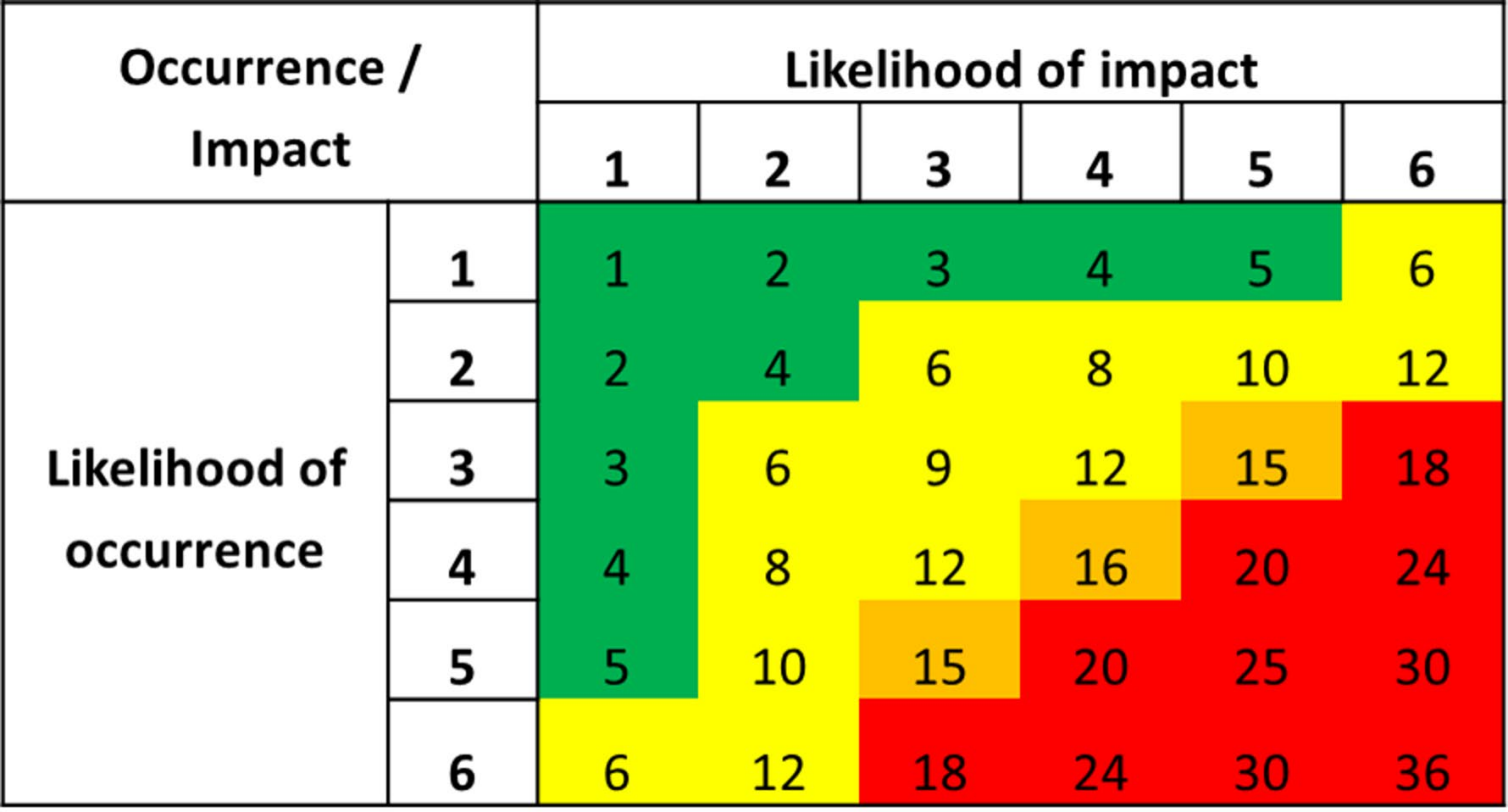
Probability matrix and impact risk.

This step is essential to develop an effective and relevant strategic plan. To identify the main risks in the scenario development, the risk levels and risk appetite data provided by the expert panel should be analyzed, i.e., the average risk should be calculated by multiplying the average occurrence and impact values, while the risk appetite index should consider the average perception, and the probability and impact matrix in Fig. 5 can be used to categorize and interpret these levels.

The risk appetite index is uniformly classified into three categories: “low” for values ​​equal to or less than 2, “moderate” between 2 and 4 (inclusive), and “high” for values ​​between 4 and 6 (inclusive). Based on the aggregation of risk levels and risk appetite values, combined with the SWOT analysis, four strategic scenarios were defined, each corresponding to one of the quadrants of the matrix. The inflection point for the risk level is set at 18, as per the probability and impact matrix illustrated in Fig. 5, while the value of 4 represents the threshold for the index of the risk appetite, marking the beginning of the “somewhat, somewhat high” category.

#### Characterization of strategic scenarios (Step 10)

The definition of strategic scenarios must consider the identified risks, the respective risk appetite, and the results of the previously performed SWOT analysis. Each scenario reflects a specific perspective on the risk categories, as well as the impact of these risks on the construction of the strategic vision.

### Risk management (Phase 4)

The implementation of strategies to reduce vulnerabilities to risk is part of risk management, as well as the position of resources to exploit opportunities and increase resilience to severe conditions^43^. The proposed risk management phase consists of two stages. The first involves selecting the relevant risks to achieve the strategic vision in each of the four scenarios, and the second consists of a critical analysis of the scenario, with the definition of mitigation plans to reduce or eliminate the identified risks.

#### Scenarios analysis to achieve the strategic vision (Step 11)

A thorough analysis of scenarios is imperative for realizing the strategic vision of any organization. Decision makers can predict challenges, recognize opportunities, and develop solid strategies to deal with uncertainty efficiently. In this matter, the output of the strategic scenarios needs to be analyzed regarding the deviation from the previous strategic vision. It is necessary to look for its fulfillment by planning measures and action plans to meet the strategic vision.

#### Critical analysis (Step 12)

This critical analysis includes the understanding of the population under study regarding, in this study, the implementation of the I4.0 and their knowledge of the I4.0 concepts. The ability to interpret the stakeholders’ perspectives is also important to meet future requirements. This population also needs a critical analysis of risk management and knowledge about the possibilities of companies receiving support for innovation. By meeting these fundamental requirements, it is possible to conduct a comprehensive and insightful analysis, identifying strengths, weaknesses, and potential areas for further investigation or improvement in the strategic vision and risk mitigation plans outlined.

## Application and testing of the methodology

Using the methodology proposed in Fig. 4, scenarios were developed that portray the main risks and opportunities faced by the industrial companies of this sample. The response to these challenges can be achieved through the implementation of structured, resilient, and appropriate strategic plans, supported by informed risk management and by promoting an environment conducive to innovation in the enabling technologies of I4.0.

### Defining the scope of scenarios

The scope of the scenarios must be in line with the companies’ strategic objectives to ensure that they are relevant and useful in supporting the decision-making process. Defining the scope of the scenarios is extremely important, as it defines the development of the methodology and is the backbone of all its stages, allowing for effective and informed analysis, preparing the population to face uncertainty, and making well-founded strategic decisions.

#### Objective of strategic scenarios

This case study aims to provide insights to companies, managers, and decision-makers to support the transition to I4.0. Strategic scenarios are used to position organizations relative to the defined strategic vision and to identify the key risks associated with I4.0 adoption, as well as how these risks can be effectively managed.

#### Definition of the time horizon of the scenarios

For the development of the strategic scenarios, given the unpredictability inherent in the long term, the year 2030-time horizon was established. This choice is justified by the relevance of the financial resources made available throughout the decade through the Portugal 2030 program and the Recovery and Resilience Plan (RRP), fundamental instruments for supporting economic and digital transformation. The Portugal 2030 program, in force since 2021, fix the national strategy for the application of European funds until 2029. At the same time, the RRP, created in response to the impacts of the COVID-19, includes strategic investments to be implemented until 2026 to promote a sustainable and resilient economic recovery.

This framework reveals several critical challenges, such as the shortage of skilled workers, the requirement for continuing education, the limited available human resources, and the need for significant investments. In this context, the year 2030 appears as a turning point, marking the end of a decade characterized by strong financial incentives and digital transformation policies. Therefore, it constitutes an appropriate and strategic time reference for the construction of the proposed scenarios.

#### Characteristics of the population under analysis

In this case study, the population analyzed was in the Setubal Peninsula, in Portugal, and corresponds to industrial companies in a region with 1,421 km² and approximately 808,689 inhabitants, according to preliminary data from the 2021 Census^49^. This area, made up of nine municipalities, is considered strategic for the installation of industries due to its privileged location, good accessibility, presence of higher education institutions in the areas of seaports, engineering and management, and near to Lisbon and its airport.

To boost regional development and attract new investments, the Associação das Empresas da Península de Setubal (AISET) was created in 2014, a representative entity that brings together 55 associated companies of different sizes, from micro to large companies^50^. AISET played a fundamental role in this case study by facilitating contact with companies in the region and allowed a better understanding of the local industrial reality.

### Strategic vision

The framework designed to support the strategic vision is primarily based on the I4.0 enabling technologies’ implementation and the definition of their respective maturity levels. To contextualize the topic among stakeholders before collecting their opinions, a PowerPoint presentation was prepared to be used in the interviews, serving as a basis for discussion.

#### Selection of representative stakeholders

To the assessment of the maturity of I4.0 in the Setubal Peninsula, it became essential to define strict criteria for the selection of experts with leadership roles in companies, business associations, higher education institutions, as well as with consolidated research experience in the area. To ensure the relevance and quality of the contributions raised, specific eligibility criteria were established: for professionals from companies or associations, it was required that they hold senior management positions in entities based in the region; in the case of researchers, they needed to present an institutional and scientific connection to the topic of I4.0, as well as knowledge of the industrial context of the Setubal Peninsula.

The selection of participants was carried out based on the researchers’ prior knowledge and their contact networks. With the support of AISET, invitations were sent by email to representatives of companies, associations, and researchers who met the defined criteria. The invitation process took place between June 17 and September 6, 2024, resulting in the confirmation of participation by all eight invitees. The detailed profiles of each stakeholder are presented in Table 2.

**Table 2:** Representative stakeholders.

Stakeholder	Role	Area of investigation/ Industrial sector
1	Executive administrator of AISET	-
2	I4.0 shop coordinator	Department of information systems and technologies
3	Dean of science and technology faculty	Research unit in mechanical and industrial engineering
4	Responsible of finance and human resources department	Industry of engineering and telecommunications
5	Executive director	Industry of graphic arts
6	Associate professor	Mechanical engineering and industrial management
7	Ex-president of The Board of Directors	Industrial start-ups
8	Commercial director	Technology information

#### Development of a basis for strategic reflection

The proposed strategic basis was based on the principles of I4.0, with an emphasis on enabling technologies and their associated maturity levels. This approach was aligned with the needs of the study and the information collection structure planned with the stakeholder panel.

Given that the concept of I4.0 maturity is still vague and somewhat complex in its definition, it has become essential to establish a clearer and more objective framework. This framework aims to guide the focus towards common aspects and characteristics that, together, allow the level of maturity to be defined. For that, it was used the I4.0 maturity definition of Schumacher et al.^51^ as *“the external or internal conditions associated with the development of vertical*,* horizontal*,* and end-to-end engineering integration”* to receive and collect opinions from the stakeholders focusing on the expected state of the three I4.0 integrations in the Setubal Peninsula in 2030. To support this process, a presentation lasting 15 min (average) was developed and used as a basis for stakeholder interviews.

#### Elaboration of the strategic vision

To collect qualitative data, the process began with an open-ended question aimed at stakeholders, which could be supplemented by other questions, depending on the participant’s profile, with the aim of obtaining a comprehensive view in the Setubal Peninsula of the I4.0 maturity by 2030. Thus, the interviews followed a semi-structured format.

Due to the iterative nature of these interviews and the unavailability of some participants, the interviews were conducted remotely, through the Zoom or Microsoft Teams platforms, between June 24 and July 26, 2024. A presentation of the strategic basis was included in each session that supported the reflection and lasted 47 min (average). Each interview was attended by two researchers: one responsible for presenting and conducting the questions, and another responsible for preparing the minutes to capture perceptions.

The stakeholders’ contributions were critically analyzed, allowing a global vision of the stakeholders’ panel. This analysis was organized in two phases: (1) verification of the opinions collected through a critical content analysis and (2) formulation of the projected vision for 2030 based on the results obtained.

As the nature of the collected data is qualitative, the use of content analysis was proposed as a method of processing and interpreting the information. This approach allows the qualitative data collected to be examined, identifying patterns, similarities, and differences in the responses, with the aim of establishing relationships and drawing conclusions relevant to the study^52^.

Based on the critical content analysis, a global vision shared by the panel was constructed regarding the level of I4.0 maturity in the Setubal Peninsula by 2030. Moreover, to define this future strategic vision, the current state of maturity of I4.0 was also characterized. This assessment of the starting point is vital to understand the degree of implementation in companies and to identify the path needed to achieve the objectives set by the stakeholders.

The aggregated view of stakeholders regarding the expected status of the three integrations by 2030 is summarized in Table 3. The responses collected during the interviews were organized and classified according to the different levels of conviction expressed by participants regarding the presence and maturity of these integrations in the time horizon considered.

In vertical integration, most stakeholders were optimistic (2, 3, 4, 5, and 6), stating that by 2030 companies will be fully integrated vertically. Another stakeholder (7) believes in integration, but points out that, vertical integration will be more informal in small companies, due to the high investments required. Finally, there are doubts and pessimism on the part of two stakeholders (1 and 8), who question the full existence of this integration or point out that many solutions are still in the evaluation phase. Based on the stakeholders’ opinions, conclusions can be drawn as follows:

• 62.5% of the opinions believe by 2030, the vertical integration will be a reality;

• 12.5% ​​of the opinions maintain an optimistic view, but say that, the vertical integration will be informal in small companies;

• 12.5% ​​of the opinions adopt a neutral position, unable to conclude on its viability;

• And 12.5% ​​of the opinions do not believe in vertical integration in companies by 2030.

Horizontal integration by 2030 presents varying levels of conviction among stakeholders. An optimistic group (2,4, and 6) believes that, in companies connected in the same value chain it will be a reality. Another group (3, 5, and 8) conditions its success on the advancement of technologies such as 5G, artificial intelligence, and cybersecurity. There are also those (7) who believe that SMEs will be pressured to integrate to retain customers, while one stakeholder (1) expressed skepticism about the existence of such integration in the expected time horizon. Based on the categorization carried out about horizontal integration, conclusions can be drawn as follows:

• 37.5% of the opinions believe that horizontal integration will be a reality by 2030;

• 37.5% of the opinions make this integration conditional on the advancement of the technologies involved, although they express optimism regarding its evolution;

• 12.5% of the ​​opinions believe that companies probably will be forced to adopt horizontal integration, but remain uncertain as to whether it will be achieved by 2030;

• 12.5% ​​of the opinions do not believe that this integration will occur within this time frame.

The responses to end-to-end engineering integration were less dispersed, allowing for the definition of fewer levels of conviction. Six stakeholders (3, 4, 5, 6, 7, and 8) demonstrated a good confidence in this implementation, emphasizing that it will encompass all stakeholders in the life cycle of the product. One stakeholder (2) did not present a specific forecast for 2030. The last stakeholder (1) adopted a more pessimistic view, considering that most companies will not yet be connected through this integration by then. Based on the categorization carried out, conclusions can be drawn as follows:

• 75% of the opinions believe that by 2030, end-to-end engineering integration will be a reality;

• 12.5% ​​of the opinions do not have a concrete opinion about this integration by 2030;

• 12.5% of the opinions ​​do not believe that such integration will occur by 2030.

Based on the results obtained, the strategic vision outlined by the stakeholder panel points to the implementation of the three types of integration in companies on the Setubal Peninsula by 2030.

**Table 3:** Stakeholder panel opinions.

Maturity	Opinions
Vertical integration	1) *“Vertical integration will be in an evolving phase*,* varying according to market dynamics and competitive pressures in the same value chain.”*.
	2) *“Vertical integration will be fully in companies*,* but as medium-sized companies will follow the evolution process*,* small companies will be facing a more challenging scenario*,* operating in survival mode. Probably*,* some small companies will adapt and will respond to the challenges of I4.0 adoption”*.
	3) *“Vertical integration will be implemented due to the CPPS interoperability*,* making companies’ production systems connected to communication systems”*.
	4) *“Vertical integration will be achieved through interconnection between machines*,* data acquisition systems*,* cloud solutions*,* and systems such as ERPs”*.
	5) *“Vertical integration will be characterized by digitalized processes*,* into cloud platforms*,* with IoT-based sensors and data analytics integrated”*.
	6) *“Vertical integration will provide internal communications and interconnections between all their elements”*.
	7) *“Vertical integration will occur in medium and large companies through ERP systems*,* while small companies will not have this level of integration and face greater challenges in meeting the dynamic demand. Even so*,* there may be cases of small companies with a higher level of vertical integration*,* through informal structures allowing the purposes of this integration”*.
	8) *“Vertical integration will not yet be fully realized*,* as the associated technologies and their benefits will remain in the evaluation phase”*.
Horizontal integration	1) *“Horizontal integration will be absent*,* and companies will not yet be interconnected with each other”*.
	2) *“Horizontal integration will exist in companies*,* enhancing this value through knowledge shared within them into the value chain”*.
	3) *“Horizontal integration will occur as an offshoot of vertical integration*,* relying on greater agility and precision in communications between companies*,* which will favor the reduction and mitigation of errors”*.
	4) *“Horizontal integration will have a higher influence in interactions between companies that exert greater influence over the organization’s core business”*.
	5) *“Horizontal integration in the downstream will be encouraged through digital trade and in the upstream through the incorporation of the supply chain*,* with constant improvements driven by artificial intelligence and reinforced by cybersecurity mechanisms”*.
	6) *“Horizontal integration will be fully in the same value chain across all elements”*.
	7) *“Horizontal integration*,* in small and medium-sized companies*,* will be facilitated by pressure from customers*,* who will have to integrate within the value chain to maintain their position as suppliers”*.
	8) *“Horizontal integration by 2030 will significantly advance thanks to the implementation of 5G technology”*.
End-to-end engineering integration	1) *“End-to-end engineering integration in most companies will not be present”*.
	2) No response provided.
	3) *“Through data sharing*,* the end-to-end engineering integration will exist*,* driven by the formation of strategic partnerships and through the needs of technological dependencies”*.
	4) *“Characterized by a higher level of strategic synergy*,* the product lifecycle will be integrated within partner companies”*.
	5) *“End-to-end engineering integration will be driven by virtual design and circular economy*,* promoting sustainability within the value chain”*.
	6) All stakeholders throughout the product lifecycle will be involved through end-to-end engineering integration.
	7) Products that involve high investments and that justify a double effort for customers are considered smart products, especially in end-to-end engineering.
	8) Companies will communicate more as the product life cycle will be shorter.

### Scenario construction

Understanding the real context of companies is crucial for developing scenarios, such as the activity, market conditions, competitors, or stakeholders. It is also important to analyze the trends (internal and external) that will impact companies. Scenarios must be realistic and challenging to stimulate innovation and anticipate future issues. Therefore, it is very pertinent to have a holistic panel of key experts, with different industry relationships, to help in developing realistic scenarios.

#### Selection of key experts

Those responsible for leadership roles in companies or associations in the Setubal Peninsula were asked to nominate professionals to participate in the study. The criteria for selecting researchers were to be affiliated with higher education institutions located in the Setubal Peninsula or nearby regions, and to have proven experience or relevant research in the areas of operations management or to develop projects with a direct link to the Setubal Peninsula industry.

Experts’ panel invitations were sent between 01/07/2024 and 22/07/2024. Of the total of 27 invitations sent, 15 were accepted, representing an acceptance rate of approximately 55.6%. Table 4 presents the profiles of the experts who make up this panel. This selection ensured a diverse and representative panel in terms of industrial sectors, organizational roles, and professional experience, strengthening the validity of the expert-based evaluation.

**Table 4:** Panel of the key experts.

Expert	Role	Principal economic activity
1	Operations director	Steel and alloy steel manufacturer
2	Finance and HR department responsible	Engineering and related technical activities
3	Executive director	Reproduction and printing of recorded media: other printing
4	General director	Metallic structures manufacturer
5	Process improvement specialist	Pulp manufacturer
6	Supply chain manager	Ink manufacturer
7	Managing partner	Brick manufacturer
8	Plant manager	Manufacturer of accessories and other components for motor vehicles
9	Coordinator	Not applicable
10	Product manager	Metallic structures manufacturer
11	Associated professor	University
12	Senior vice president operations and supply chain	Automotive electronic components
13	IT manager	Metallic automotive components
14	Commercial director	Metallic structures
15	Maintenance director	Wine manufacturer

#### Identification of risks and risk categories

The experts were invited to reflect on the identified risks, indicating their degree of agreement regarding their existence, classifying them according to the SWOT matrix, and evaluating them based on the three proposed risk parameters. To this end, fifteen remote interviews were conducted between July 8, 2024, and July 26, 2024, through the Zoom or Microsoft Teams platforms, with 52 min each (average duration). The interviews were conducted by two researchers: one responsible for leading the interview and the other responsible for systematically recording the responses. With the support of the Microsoft Excel tool, the information was aggregated, processed, and analyzed.

#### Key risks selection

**Fig. 6:**
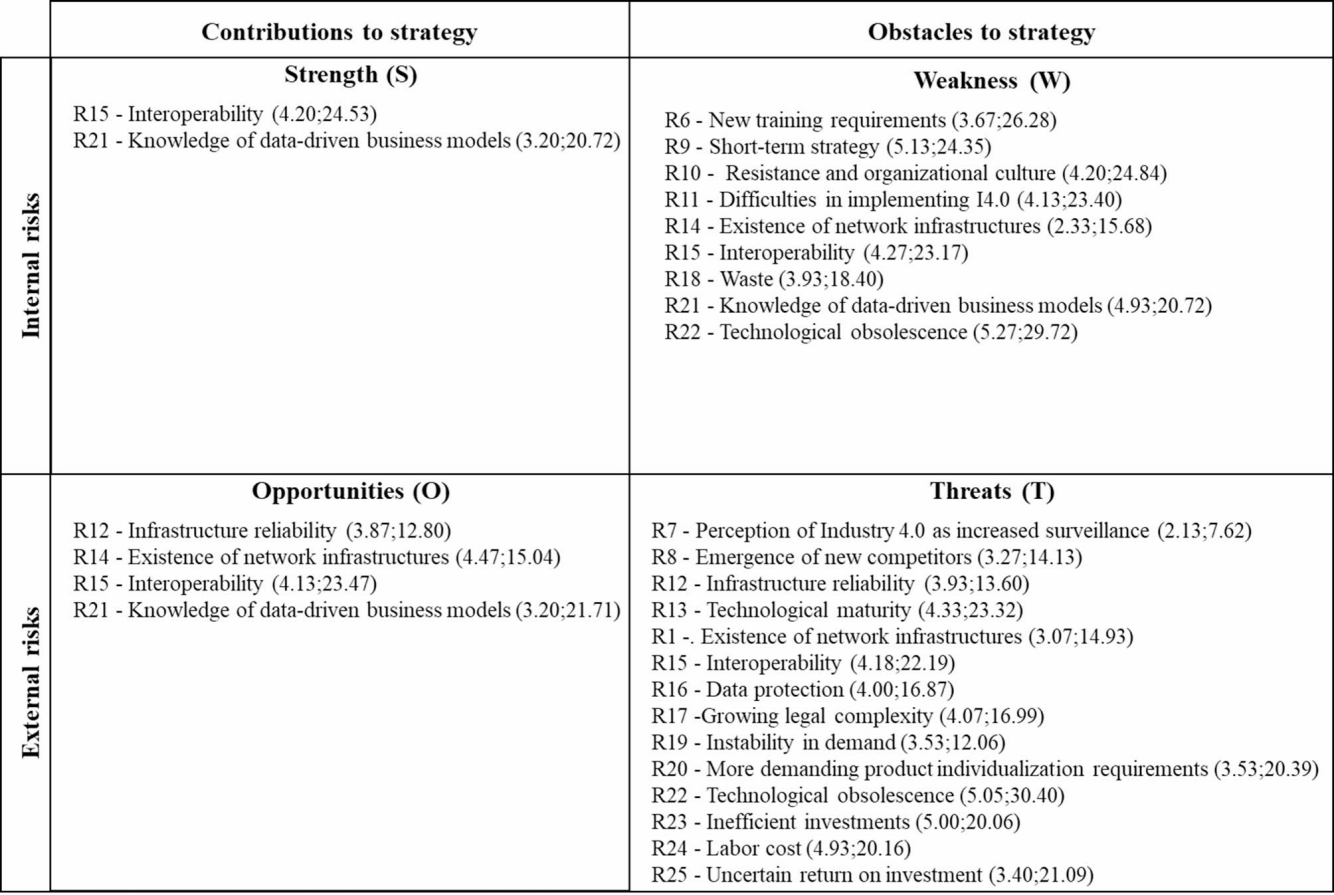
Risks identified and validated by key experts.

The results of the experts’ main assessments and validation were integrated into the risk list analysis, complementing the Risks (R) collected in the literature and the Risk Categories (RC) established in Table 1. The final version of the list, presented in Fig. 6, brings together the average risk appetite index, the values of the average risk level, and the SWOT analysis validated by the different experts consulted.

To provide qualitative interpretations of inter-rater reliability estimates, it is important to show that respondents can independently reach similar conclusions about the items observed^53^. For assessing the reliability of agreement between fixed raters when evaluating items, the Fleiss’ Kappa coefficient can be used with an ordinal scale^53,54^, as the Likert scale^53^. Several studies have used the same approach. Fleiss (1971)presented a study with 6 raters and thirty evaluations with a 1 to 5 scale. Hoelbling et al.^55^ used Fleiss’ Kappa in two samples of six and sixteen raters. Lughbi et al.^56^ used three raters to evaluate data. Chatzipetrou et al.^57^ used three raters to assess the quality of natural language requirements.

This case study used a sample of fifteen experts (raters) of Table 4, with thirty-five observations (risks of Fig. 6), on a Likert scale of 1 to 6. The calculated Fleiss’ Kappa of the occurrence, impact, and risk appetite is shown in Table 5, also with significance tests, standard error, and confidence interval values.

**Table 5:** Values of inter-rater reliability.

Values	Occurrence	Impact	Risk appetite
Fleiss’ Kappa	0.450	0.463	0.445
Standard Error (SE)	0.00840	0.00877	0.00880
z-Value	53.54	52.80	50.50
Confidence Interval (CI 95%)	[0.433;0.466]	[0.446;0.480]	[0.427;0.462]

Different interpretations have been suggested in the literature for assessing the strength of agreement between raters. In this case study, the interpretation follows the guidelines of Landis and Koch^58^. Assuming the interpretation of Table 6, the results indicate that the data collected has a moderate strength of agreement between experts, as shown by the interpretation of Fleiss’ Kappa. The significance tests show that k differs significantly from zero, with z higher than the critical value of 1.96, with a significance level of 0.05. This indicates that the observed agreement is significantly greater than expected by chance. The confidence intervals for k provide a measure of the accuracy of this estimate. These results can be used to evaluate the reliability of agreement between the fifteen raters on the thirty-five items. Corrections and revisions to the standard error calculations ensure the validity of the analysis.

**Table 6:** Interpretation of Fleiss’ Kappa values (Landis & Koch, 1977).

Fleiss’ Kappa statistic	Strength of agreement
< 0.00	Poor
0.00–0.20	Slight
0.21–0.40	Fair
0.41–0.60	Moderate
0.61–0.80	Substantial
0.81–1.00	Almost perfect

**Fig. 7:**
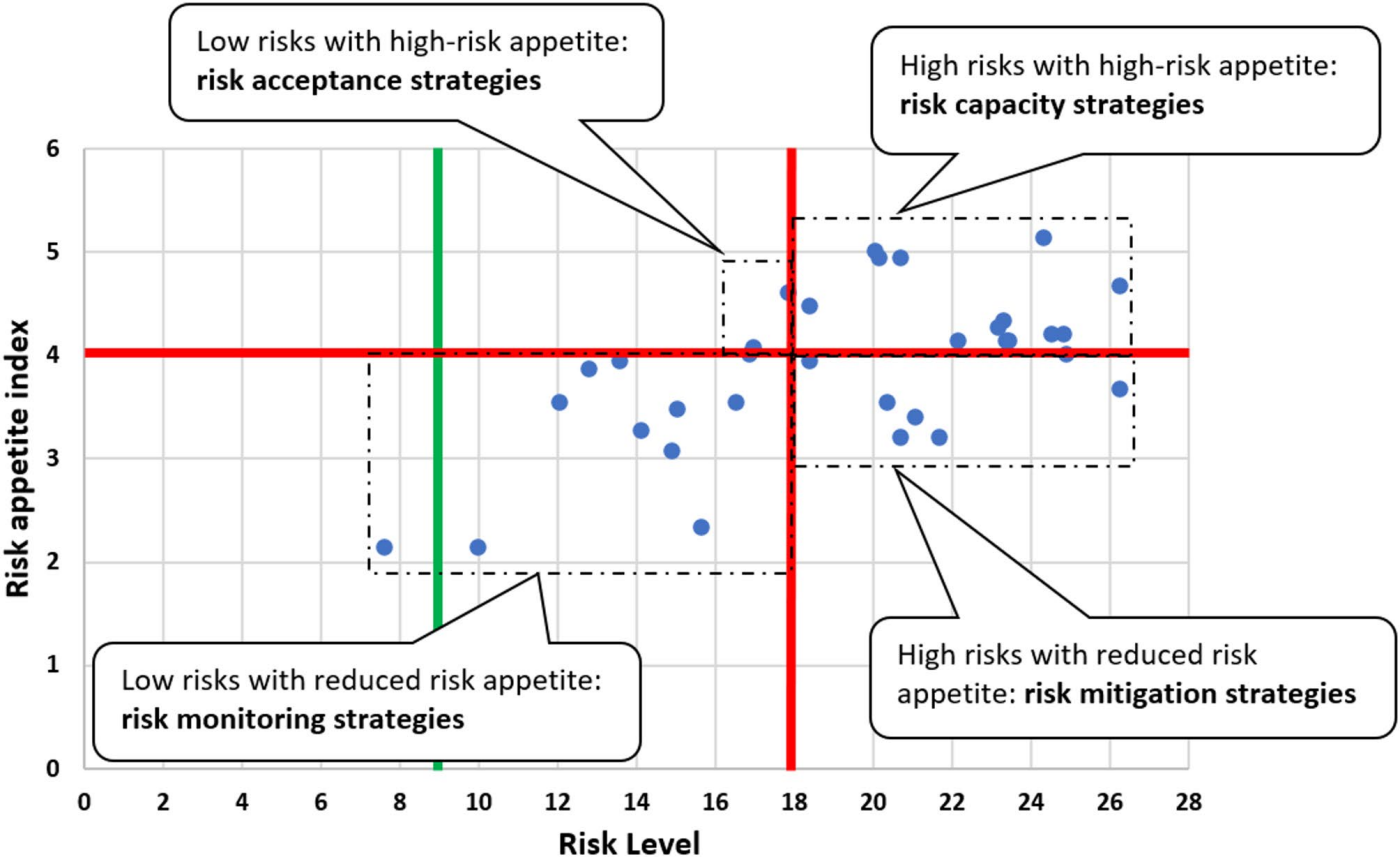
Analysis of the key risks.

It is essential to consider both the risk level and the risk appetite index to define strategic scenarios. Risks with a high appetite require strengthening the internal capacity of organizations, while risks with a low appetite and high impact are considered critical and should be targeted by mitigation strategies. Figure 7 presents the four strategic scenarios derived from the combination of risk level and risk appetite. Each quadrant represents a distinct strategic positioning, while the plotted risks illustrate how individual risks were distributed within these scenarios, using threshold values of 18 for the risk level and 4 for the risk appetite index. This visualization enables a clearer comparison between scenarios by highlighting the relationship between aggregated risk exposure and organizational risk tolerance, supporting the definition of corresponding strategic responses.

The answer of the RQ3 are the risks identified to be mitigated as R6 - New training requirements (W), R18 - Waste (W), R20 - More demanding product individualization requirements (T), R21 - Knowledge of data-driven business models (S), R21 - Knowledge of data-driven business models (O) and R25 - Uncertain return on investment (T).

During the interviews with the key-experts panel, when evaluating risks identified in the literature, it aroused some new risks that were not considered in this case study, namely, cyber blackmail, departure of workers to other companies, talent retraining, talent hiring, adequacy of labor legislation to the new productive reality, international labor integration collaborations protocol, emergence of innovative products, products and services with innovative features, unfair competition, evolution of competition, agility and flexibility of the organizational structure, incorporation of stakeholders into the value chain of the organizational structure, difficulty in finding solutions for infrastructure, changes in fees and taxes, valorization of surpluses, reduction of energy consumption, online transactions, growing market demand, liquidity/treasury management, and impersonalization between customers and suppliers. Nevertheless, it is important to mention those risks for further studies.

#### Characterization of strategic scenarios

“Acceptance Strategies” is the first scenario projected for 2030, characterized by low risks and high-risk appetite. Focused exclusively on external risks (Threats), this scenario adopts a more defensive perspective, focusing on factors external to organizations. Companies demonstrate a willingness to face relevant challenges and take significant risks, intending to seize the opportunities associated with digital transformation. This scenario is characterized by a proactive approach, where companies recognize the risks associated with I4.0 adoption but are determined to overcome them through acceptance strategies. A crucial part of this strategy is awareness and preparation for identified risks. In the case of concerns about the disappearance of jobs (R4), companies are investing in employee retraining and retraining programs, ensuring that they can adapt to the new demands of the digital labor market. Additionally, they are exploring collaboration models between humans and machines to maximize efficiency and innovation. Regarding data protection (R16) and the increasing legal complexity (R17), companies are taking a proactive compliance stance.

These risks influence the strategic vision with significant investments in cybersecurity systems and regulatory compliance programs such as GDPR (General Data Protection Regulation) in the European Union. They are also actively participating in standard-setting and regulatory initiatives to ensure that I4.0 is developed and implemented ethically and safely. Despite these challenges, companies have assessed the risks associated with the I4.0 implementation as having a low level of probability of occurrence, while at the same time having a high appetite to assume these risks. This suggests they recognize the obstacles but are confident in their ability to mitigate them and are committed to innovation and digital transformation. In the strategic scenario of “acceptance strategies” for the I4.0 implementation, companies are adopting a proactive approach to face the associated risks, investing in human and technological resources to ensure a smooth transition to the new industrial era.

“Monitoring Strategies” is the second scenario projected for 2030, associated with low levels of risk and a reduced risk appetite. In this scenario, negative external factors (Threats) predominate. Companies that analyze I4.0 adoption from this perspective tend to identify risks and opportunities that may compromise the viability and effectiveness of their strategies. Critical threats include industrial espionage (R2), given the increase in connectivity and data sharing that exposes companies to theft of sensitive information, and industrial sabotage (R3), which also represents a significant risk. Connected and automated systems are vulnerable to attacks that can compromise the integrity of processes and cause substantial damage. I4.0 may increase the perception of surveillance among employees (R7), which is seen as a threat. This feeling can affect morale and productivity and cause resistance to adopting new technologies. The emergence of new competitors (R8) is a threat, as I4.0 facilitates the entry of new players into the market, especially those that are technologically advanced and agile. Infrastructure reliability (R12) is seen as both an opportunity and a threat. While reliable infrastructures are essential for the success of monitoring strategies, their lack or failures can compromise the entire system. The existence of network infrastructures (R14) is simultaneously an opportunity, weakness, and threat. The availability of a robust network is crucial to supporting I4.0 operations, but dependence on this infrastructure also poses a significant risk in the event of failures or attacks. Instability in demand (R19) is a threat, as volatile market conditions can affect the consistency and predictability of operations and investments required to implement I4.0.

The influence of these risks on the strategic vision indicates a cautious stance towards adopting new technologies, prioritizing risk mitigation, and ensuring security and stability in operations. To successfully implement I4.0 monitoring strategies, companies must develop robust cybersecurity plans, communication strategies to manage employee perceptions, market analysis to predict and adapt to competition and demand, and ongoing investments in trusted infrastructures. A careful approach and proactive management of identified risks will help maximize the benefits of I4.0 while minimizing potential threats.

The third scenario projected for 2030 falls within the “Mitigation Strategies” category, characterized by high levels of risk and low risk appetite. In this context, I4.0 adoption represents a challenging process, requiring rigorous management of the risks involved to ensure an effective and sustainable transition. In the new training requirements (R6), the need for new knowledge and skills among workers is a significant challenge. The transition to I4.0 requires employees to acquire skills in advanced technologies such as artificial intelligence, big data, and automation. Lack of adequate training can result in a skills gap, affecting productivity and the effectiveness of operations. The introduction of new technologies and processes may initially lead to an increase in waste (R18) of materials and energy. Without a robust plan to manage and reduce this waste, companies can face high operating costs and negative impacts on the environment, compromising their sustainability goals. In the more demanding product individualization requirements (R20), the growing demand for personalized products imposes additional challenges on companies. Adapting to these requirements requires investments in flexible technologies and adaptable production processes, which can be financially costly and operationally complex. In knowledge about data-driven business models (R21), the ability to use data to develop new business models is both a strength and an opportunity. Companies that can leverage data to optimize their operations and create new services can gain a significant competitive advantage. However, successful implementation requires investments in data infrastructure and analytical skills. The high initial cost to implement I4.0 technologies and uncertainty about the return on investment (R25) represents a substantial risk. Companies may be hesitant to invest without clear guarantees that these investments will result in tangible, measurable benefits in the medium and long term.

The influence of key risks on the strategic vision involves continuous training, training, and development programs to ensure that workers acquire the necessary skills to operate new technologies, correct waste management, and the implementation of quality and efficiency control processes. to minimize waste and improve sustainability, in production flexibility with investments in technologies and processes that allow mass customization efficiently and economically. Data infrastructure is also very important in fulfilling the strategic vision, of developing a robust data infrastructure and analytical capabilities to transform data into valuable insights. Finally, ROI assessment using rigorous methodologies to evaluate the return on technology investments, with continuous monitoring of results to adjust strategies, as necessary.

The fourth scenario predicted for 2030 corresponds to “Capability Strategies”, characterized by a high level of risk and a high appetite for risk. This scenario is predominantly influenced by adverse internal factors (Weaknesses), followed by external threats. “Capability Strategies” require a robust strategic approach, capable of dealing with multiple dimensions of risk that directly impact decision-making and organizational planning. Identified as a threat in the SWOT analysis, the loss of sensitive information (R1) stands out due to its potential vulnerability to cyber-attacks that could compromise critical company data. Given the sensitive nature of the information managed in I4.0, this risk is assessed at a high level of risk, requiring robust cybersecurity measures. The lack of skilled labor (R5) and new training requirements (R6) are classified as threats. The transition to I4.0 demands a workforce with specific skills in advanced technologies. The shortage of qualified professionals and the need for ongoing training represent significant challenges. As weaknesses, the short-term strategy (R9), resistance and organizational culture (R10), and difficulties in I4.0 implementation, are risks that reflect the inadequacy of current organizational strategies to support long-term technological transformations, resistance internal to changes and the traditional organizational culture that can hinder the adoption of new technologies. Technological maturity (R13), assessed as a threat, can limit companies’ ability to integrate new technologies effectively. Interoperability (R15) is simultaneously seen as an opportunity, weakness, and threat, being crucial for the integration of systems and processes, but the lack of it can create significant obstacles. If well managed, it can be a competitive advantage, but inefficiency can compromise the operation. Knowledge about data-based business models (R21), as a weakness, can be assumed to be the lack of understanding about how data can be used to create new innovative business models, limiting the transformation potential of companies in I4.0. Rapid technological evolution can make recent investments obsolete in the short term, imposing the need for continuous reinvestment, resulting in technological obsolescence (R22). Inefficient investments (R23) can result in lower-than-expected returns, while high skilled labor costs (R24) pose a significant financial challenge.

The influence of these risks in strategic vision comprises the high level of risk due to its potential significant negative impact on companies’ operations and strategies. However, the high-risk appetite indicates the willingness to invest and advance in the implementation of I4.0, even in the face of challenges. This provision reflects the understanding that, despite the high risks, the potential benefits in terms of efficiency, innovation and competitiveness can outweigh the threats, provided that the risks are managed effectively with proactive strategies and appropriate investments.

### Risk management

In risk management, by integrating critical analysis into this process, professionals can identify not only strengths and opportunities, but also potential challenges and risks associated with each scenario. This critical approach is essential to ensure that the adopted strategy is based on a deep understanding of the business environment and can help with the uncertainties and variability that may arise. Critical analysis also allows for continuous review of scenarios, ensuring that strategies remain aligned with organizational objectives and are adjusted as necessary to respond to changes in the external environment. In this way, the combination of scenario analysis and critical analysis provides companies with a solid foundation for developing and implementing strategies that drive long-term success.

#### Scenarios analysis to achieve the strategic vision

In the first scenario “Acceptance Strategies”, companies show the intention to invest in the transition to I4.0, facing risks considered low. However, to reach the strategic vision, it is essential that organizations recognize and work on the various critical elements that may still be missing in this optimistic context. Companies must develop an advanced technological infrastructure with the implementation of high-speed networks, such as 5G, to support real-time communication between devices and systems, with the widespread adoption of smart sensors and IoT devices to collect and Process data across the entire value chain. The development and adoption of platforms that integrate data and processes from all levels of the company, from the factory floor to administration, are key factors for an advanced technological infrastructure. Continuous training and skill development programs in digital technologies, automation and data analysis and the promotion of a culture of innovation and adaptation, where employees are prepared to work in highly digitalized environments are factors that strengthen the training and requalification of the work force. Implementing robust cybersecurity solutions to protect sensitive data and ensuring system integrity and developing rigorous data protection policies and compliance with international regulations enhance cybersecurity. Government collaboration can be achieved by working together with regulatory authorities to develop and update regulations that keep pace with technological developments. Industrial standards can be facilitated with interoperability and integration between different systems and platforms. For companies, there is access to financing for investments in new technologies and infrastructure necessary for digital transformation and incentive policies, such as subsidies and tax benefits, to encourage the adoption of I4.0 technologies. When sharing knowledge, it is essential to develop ecosystems that promote collaboration between companies, universities, research centers and startups, as well as strategic partnerships to share resources and technologies. In management and strategy, committed and visionary business leaders are needed to drive digital transformation, with change management plans that ensure a smooth transition to new operational and business models. By focusing on these objectives, companies can create a clear and successful path to achieving the strategic vision of 2030, where vertical, horizontal, and end-to-end engineering integrations are fully implemented, resulting in more efficient, innovative operations and competitive.

In the second scenario “Monitoring Strategies”, companies are reluctant to invest significantly in the transition to I4.0 due to their risk aversion. For the strategic vision to come to fruition, these organizations will need to take complementary actions and promote additional developments that mitigate their concerns. It is necessary to improve and expand communication networks. It is crucial to invest in high-speed, low-latency communication networks, such as 5G and future connectivity technologies, to ensure efficient interconnection between all levels of the production chain. Modernizing IT systems to support large volumes of data and guarantee the security and integrity of information exchanged between different levels of integration is essential for a robust technological infrastructure. Establishing rigorous cybersecurity protocols to protect against espionage and industrial sabotage includes the adoption of technologies such as artificial intelligence for anomaly detection, end-to-end encryption, and advanced firewalls in the action plan. The constant training of IT and cybersecurity teams to deal with emerging threats and maintain a safe environment makes cybersecurity reinforced. To engage employees, it is necessary to develop training and engagement programs to reduce the negative perception of surveillance and increase acceptance of new technologies among employees, as well as foster an organizational culture that values innovation and rapid adaptation to technological changes. Interoperability and standardization are very important. Implementing industrial standards to ensure interoperability between different systems and platforms used in vertical and horizontal integration and developing solutions that facilitate the integration of legacy systems with new technologies, avoiding the obsolescence of previous investments, is mandatory. Companies must establish partnerships with technology providers, universities, and research centers to accelerate the development and implementation of innovative solutions and promote collaboration with suppliers, distributors, and other stakeholders to ensure effective horizontal integration aligned with strategic objectives. Systems for continuous monitoring of market conditions and competitor activities must be implemented to adapt business strategies in an agile manner and develop flexible business models that can quickly adapt to changes in market demands and economic conditions. Companies must continue to invest in research and development to innovate in automation technologies, IoT, AI, and other areas crucial to I4.0. They must also use agile methodologies to prototype and test new technologies and processes before their large-scale implementation. Companies must work with governments and regulatory entities to create favorable incentive policies and regulations that support digital transformation and I4.0 implementation and ensure that all implementations comply with existing standards and regulations to avoid legal and operational issues. For companies to achieve the strategic vision of having vertical, horizontal, and end-to-end engineering integrations fully implemented by 2030, a coordinated effort on several fronts is essential: technological, organizational, collaborative, and regulatory. Continuously investing in technology, security, training, a culture of innovation and strategic partnerships will be key to turning this optimistic vision into reality.

In the third scenario “Mitigation Strategies”, companies recognize the risks associated with the transition to I4.0 but have limited appetite to take them on. To reach the strategic vision, companies must implement continuous training programs, allowing employees to acquire and update skills in emerging technologies, such as automation, artificial intelligence, and data analytics. In addition, companies must develop strategies to attract specialized talent in critical areas and retain professionals who are already qualified, thus ensuring a team prepared for the challenges of digital transformation. Continuous investments should be considered in the modernization of existing equipment and systems to support I4.0 technologies and focus on system interoperability, that is, ensuring that all implemented systems and technologies are interoperable and can communicate efficiently with each other, both internally and externally with partners and suppliers. Data management must focus on the development of a robust infrastructure for collecting, storing, and analyzing large volumes of data in real-time, ensuring cyber security with the implementation of measures to protect sensitive data and guarantee the integrity and confidentiality of operations digital. Continuous R&D investments must exist to maintain competitiveness and innovation, allowing rapid adaptation to new technologies and market trends, with organizational agility based on the development of a culture that allows a rapid response to changes and the implementation of new technologies and processes. Companies must invest in the development of an ecosystem of technological partners, suppliers, and research institutions that can collaborate and support the implementation of I4.0 m, as well as encourage collaboration between different departments of the company to ensure an integrated and cohesive approach. Sustainability must be guaranteed by implementing sustainable practices to minimize waste and optimize the use of resources, aligning with environmental goals, in the continuous improvement of operational processes to increase efficiency and reduce costs. Exploring new business models based on data and digital technologies creates additional value and new sources of revenue. Companies must leverage capabilities to meet demand for personalized and flexible products, quickly adapting production lines as needed. Rigorous methods for evaluating and monitoring the ROI of new technologies, and ensuring that investments generate tangible benefits, are mandatory requirements. There must be adequate financing structures to support the necessary investments in technology and innovation. Effective internal and external communication strategies are needed to ensure that all stakeholders are aligned with the vision and objectives of digital transformation and initiatives to engage employees in the transformation process, promoting a culture of innovation and continuous improvement. To achieve the strategic vision of 2030, where vertical, horizontal, and end-to-end engineering integrations are innovative, companies must address these areas in a proactive and integrated way. The combination of workforce empowerment, robust technology infrastructure development, cybersecurity, continuous innovation, strategic partnerships, sustainable practices, new business models, sound financial planning, and effective communication will create an enabling environment for realizing this optimistic vision.

In the fourth scenario “Capability Strategies”, companies are willing to invest in the transition to I4.0 and have the appetite to take on the risks involved. To reach the strategic vision, companies must address certain gaps and overcome outstanding challenges. It is essential to develop and implement advanced cybersecurity measures to protect against the loss of sensitive information. Investments in data protection technologies, continuous threat monitoring, and employee training in security practices are essential. It is necessary to implement continuous training and skills development programs to overcome the lack of qualified labor. Partnerships with educational institutions and internal training programs focused on I4.0 technologies are necessary to prepare the workforce. Companies must promote an organizational culture open to innovation and change. This involves communicating the benefits of I4.0, involving employees in change processes, and creating incentives for the adoption of new practices and technologies. They must develop long-term organizational strategies that support the ongoing implementation of I4.0, avoiding the trap of short-term strategies that may be insufficient. There must be an investment in emerging technologies and the continuous updating of technological infrastructures to ensure that technological maturity is aligned with the objectives of I4.0 and work on the standardization of systems and processes to ensure interoperability between different platforms and technologies. This may involve adopting international standards and collaborating with technology providers. The exploration of innovative business models is necessary. Encouraging research and development of new data-based business models, using big data analysis and artificial intelligence to create more efficient and personalized business strategies are approaches that companies should follow. Carrying out efficient investment management and ensuring that resources are directed to areas that will provide the highest return on investment is crucial. This includes avoiding technological obsolescence through continuous assessment of technological and market needs. Companies must implement strict controls to manage labor costs and other operational costs, always seeking efficiency and optimizing resources. The IT and OT (Operational Technology) infrastructure must be ensured so that it is adequate to support the necessary integrations. This includes robust communications networks, efficient data storage and processing systems, and automation support infrastructure. Collaboration between different companies, technology providers, research institutions, and governments must exist to create a support ecosystem that facilitates the implementation of I4.0. To achieve the 2030 strategic vision, companies need to address these critical areas in a coordinated and proactive way. The focus should be on building a solid foundation in terms of security, skills, organizational culture, infrastructure, and innovation. With well-defined strategic planning and the willingness to face challenges, the complete implementation of vertical, horizontal integrations and end-to-end engineering will be viable, allowing companies to reap the full benefits of I4.0.

#### Critical analysis

The characterization of the companies on the Setubal Peninsula was essential to understand the starting point for the objective established in the strategic vision defined by the representative stakeholders. Based on this, the strategic vision for 2030 was developed. Based on the strategic vision, risk mitigation measures were proposed, aimed at supporting companies in reducing risks and increasing their chances of reaching the needed maturity level.

During the formulation of the strategic vision, one stakeholder stated that “integration will be achieved as a result of vertical integration (…)”, when asked about the existence of horizontal integration in 2030. Considering the opinions of stakeholders in the Setubal Peninsula and the strategic focus of this study, vertical integration will be the target of the proposed risk mitigation plans, as it constitutes the first barrier in the transition to I4.0.

Based on the forecasts for vertical integration in 2030 and performing an inverse analysis, it can be concluded that the statements collected indicate that the expectations of stakeholders point, on average, to a level at which vertical integration in companies in the Setubal Peninsula will no longer be a limitation. Therefore, to accelerate and qualify progress towards the strategic vision defined by stakeholders, it will be essential to develop mitigation plans for the risks identified in the “Mitigation Strategies” scenario.

The risk “new training requirements” (R6) was classified by experts as extremely serious, i.e., red level. However, it is acknowledged that employee education and training have positive effects on all identified critical risks. This duality can be explained by two hypotheses: either key experts doubt the effectiveness of training, or they consider that the necessary resources to implement it do not exist, the latter being the most likely. Therefore, companies in the Setubal Peninsula must integrate training into their strategic plans, using European Union funds and support from Portugal 2030. I4.0 requires specific technical skills, such as AI, IoT, big data, and cybersecurity, whose acquisition requires significant investment and faces a shortage of qualified professionals in the market.

The implementation of new technologies can initially increase waste levels due to failures in systems adaptation and integration. Errors in production, inadequate machine configuration, and lack of synchronization between processes can result in significant losses of resources. Mass customization requires flexible and highly responsive systems capable of meeting specific customer demands without compromising efficiency and economies of scale. This can put pressure on production capacity and increase operational complexity. Moving to data-driven business models requires a solid understanding of big data analytics, machine learning, and artificial intelligence. However, many companies still lack the skills needed to interpret the data collected and convert it into useful information for decision-making. Adopting these emerging technologies requires significant investments in infrastructure, systems, and human resources training. The uncertainty regarding the financial return on these investments represents a significant barrier, especially for small and medium-sized companies.

The success of vertical integration depends on effective coordination within the different levels of the organizational structure. The lack of adequate training can result in knowledge gaps that make this coordination difficult. Waste and complex customization can disrupt the fluidity of vertical processes, increasing the need for rigorous monitoring and control systems. Horizontal integration requires close collaboration between different departments and external partners. Lack of familiarity with data-driven business models can compromise efficient communication and make it difficult to improve operational processes. Uncertainty about ROI can lead to reluctance to invest in the collaborative, interoperable systems required for this integration. End-to-end engineering integration involves managing the entire product life cycle. Individualization requirements increase complexity, necessitating highly adaptable and interconnected systems. Inadequate training and a lack of data analytics expertise can limit the ability to optimize each phase of the lifecycle, from design to maintenance.

Beyond the qualification of employees, the opportunity provided by programs such as the multiannual financial framework and Portugal 2030 also encompasses investment in technologies that accelerate interoperability in companies. Applications for incentives under the RRP and Portugal 2030 represent a lever to strengthen the competitiveness of companies in the Setubal Peninsula. However, access to these resources depends on the coherence between their strategic plans and the European Union’s vision for the coming years, requiring alignment with funding programs and a long-term planning approach. Table 7 summarizes the main mitigation measures and plans defined in the “Mitigation Strategies” scenario.

**Table 7:** Proposed risk mitigation plans for the “Mitigation strategies” scenario.

Risk category	Risk	Proposals for risk mitigation plans
RC2 - Labor	R6 - New training requirements	• Launch of employee training and development programs by using the initiatives and structural funds from Portugal 2030; • Expand the workforce and strengthen the retention of current professionals by the improvement of working conditions; • Hiring specialized consultants to help fill knowledge gaps temporarily; • Develop continuous internal training programs, with specific modules on new technologies and their applications; • Collaboration within research centers and universities to create education and certification programs aligned to the needs of I4.0; • Implement online learning platforms that offer up-to-date courses on emerging technologies and best practices; • Establish mentoring programs where experienced employees can guide new talent, facilitating the transfer of knowledge and skills; • Recruit professionals with experience in I4.0 technologies to lead initiatives and train the team.
RC7 - Sustainability	R18 - Waste	• Take an incremental approach, starting with pilot projects, to help identify and fix problems before full-scale implementation; • Adopt Lean Manufacturing principles through I4.0 to identify and eliminate waste in production processes. • Promote the Kaizen culture through I4.0, encouraging continuous improvement and proactive identification of problems. • Detect and correct quickly any anomalies or deviations in the production processes by using real-time monitoring systems; • Implement predictive maintenance programs to avoid unplanned downtime and minimize waste related to equipment failures; • Perform root cause analyses to understand and correct the fundamental causes of recurring waste.
RC8 - New business models and market conditions	R20 - More demanding product Individualization requirements	• Use additive manufacturing and modular production technologies to increase flexibility; • Implement integrated supply chain management systems and use optimization algorithms to help address the complexity of customization; • Achieve customization without compromising efficiency by investing in flexible manufacturing technologies, such as modular manufacturing cells and 3D printing; • Adopt agile methodologies to manage product development projects, allowing quick adjustments as necessary; • Use digital platforms and digital twins to simulate and optimize personalization processes before actual production; • Implement advanced supply chain management systems that guarantee the availability of customized materials according to customer needs; • Involve clients in the design process to ensure their specifications are clearly understood and met efficiently.
RC8 - New business models and market conditions	R21 - Knowledge of data-driven business models	· Collaborative strategies within university institutions and companies; · Investment in employee skills through training and development; · Use of incentive programs as a mechanism to promote the strategic alignment of companies in the Setubal Peninsula with the vision for digitalization of the European Union; · Adopt business intelligence and analytics tools to facilitate data intuitively with its collection, analysis, and visualization; · Recruit experienced data scientists and analysts who can lead data-driven initiatives and develop predictive models; · Promote an organizational culture that values data-based decision-making, encouraging all levels of the company to use insights derived from data; · Collaboration with technology companies and specialized consultancies to implement advanced analytics and machine learning solutions.
RC9 - Financial	R25 - Uncertain return on investment	· Use clear metrics to measure ROI and adjust strategies as necessary to ensure a positive return; · Conduct detailed cost-benefit analyses for each technology initiative, considering all potential costs and benefits; · Implement pilot projects to test new technologies on a smaller scale before full implementation, allowing for more accurate adjustments and evaluations; · Define clear key performance indicators and measure ROI on an ongoing basis, adjusting strategies as necessary; · Explore financing opportunities, subsidies, and tax incentives offered by governments and institutions for technological innovation projects; · Develop risk management plans that identify and mitigate potential financial problems related to the implementation of new technologies.

## Discussion

The objective of the proposed methodology to develop strategic scenarios was validated and the four scenarios were developed. Each scenario offers a distinct approach to risk management and opportunities associated with I4.0 adoption. The challenge is to achieve an appropriate balance between the introduction of innovations and risk control, adjusted to the reality and particular goals of each company. While some may prefer a cautious and incremental approach, others may opt for a more ambitious and proactive strategy. Ultimately, success in the I4.0 era will depend on the ability to adapt, innovate, and effectively manage risks in a rapidly evolving digital landscape. The proposed methodology outlines four different strategic scenarios that companies may adopt regarding I4.0 adoption, each with its approach to risk management and investment.

Each scenario recognizes the importance of the transition to I4.0 and the need to address the opportunities and challenges I4.0 adoption. Regardless of the approach chosen, all scenarios consider the importance of risk management during the transition to I4.0. This reflects a shared understanding that the move to I4.0 involves uncertainties and potential challenges that need to be considered and mitigated.

Scenarios vary in their approach to risk. While the “Acceptance strategies” scenario adopts a more cautious stance and seeks to minimize risk, other scenarios such as “Capacity strategies” demonstrate a greater willingness to take risks in search of growth opportunities. The scenarios differ in the extent to which they emphasize innovation. While some, like the “Capability strategies” scenario, encourage innovation and I4.0 enabling technologies adoption, the “Monitoring strategies” scenario may be more hesitant to adopt risky innovations.

Choosing the most appropriate scenario will depend on each company’s specific circumstances, including its risk appetite, strategic vision, and internal capabilities. Moreover, all approaches recognize the importance of the transition to I4.0 and the need to carefully manage the risks and opportunities associated with it. The approach with this proposed methodology differs from other methodologies found in the SLR of Appendix A, which does not include risk management to develop scenarios. The output from this methodology is four strategic scenarios in which companies can position themselves and take the necessary actions to maintain competitiveness to face market demand and competition.

From a theoretical perspective, the proposed framework can be further interpreted through the lenses of dynamic capabilities and digital maturity. The ability of organizations to sense, seize, and reconfigure resources in response to I4.0 risks is closely related to their positioning across the proposed strategic scenarios. In this context, scenarios such as “Monitoring” and “Capability” reflect different levels of digital maturity and organizational readiness to manage uncertainty and technological change.

Moreover, the four strategic scenarios should not be interpreted as static configurations but as dynamic states. Organizations may transition between scenarios over time as a result of changes in external conditions, technological advancements, or internal capabilities. For instance, shifts in risk exposure, increased digital competencies, or changes in strategic priorities may act as tipping points, enabling a transition from a “Monitoring” strategy to a more proactive “Capability” approach. This dynamic perspective enhances the strategic relevance of the framework by incorporating an evolutionary view of risk and decision-making.

Finally, although the framework is developed within a manufacturing context, its underlying logic can be extended to other sectors facing digital transformation challenges, such as healthcare, energy, and education. In these contexts, the interaction between technological innovation, risk management, and organizational capabilities remains critical, suggesting the broader applicability of the proposed approach.

## Conclusions

Strategic scenarios are an essential tool to support companies in understanding the impacts of existing risks in the sector, which can significantly influence I4.0 adoption, highlighting the possible consequences arising from their strategic decisions. Based on the assessment of a regional industrial sector, considering the risks associated with I4.0 transition, RQ1 emerged, which was answered through the artifact developed in this study: the methodology proposed for the development of strategic scenarios.

In this case study, the strategic vision outlined points to the implementation of the three types of integration by 2030. In response to RQ2, twenty-five risks were identified and positioned in the SWOT analysis, grouped into nine main risk categories. From this analysis, four distinct strategic scenarios for 2030 were characterized, each with different approaches to risk management, levels of exposure, and risk appetite. These scenarios can serve as a reference for companies to guide themselves and effectively prepare for the transition towards I4.0. Even so, it is recommended that each company use the methodology proposed in this study to understand its position more accurately in the face of the four scenarios identified. Finally, the answer to RQ3 was found in the risks associated with the “Mitigation Strategies” scenario, which requires an increase in risk appetite on the part of organizations.

The findings of this case study indicate that companies in the Setubal Peninsula should work with these opportunities offered by financial programs. These financing instruments will enable companies to align their objectives with the European Union’s long-term strategy.

The first study limitation is the small sample size used to develop the strategic vision, as well as the limited amount of data collected from relevant experts. This limitation also includes the limited number of professionals who participated in the SWOT analysis validation and the risk identification and assessment process, which compromised the performance of more detailed qualitative analyses of risks and strategic scenarios. The second limitation deals with the absence of concrete and quantified strategic objectives during the vision definition phase. The lack of these elements prevented the development of more robust and measurable strategic scenarios. For future studies, it is recommended to conduct in-depth interviews with stakeholders to collect specific and quantifiable objectives, or to extract such objectives from the incentive guidelines of the European Union or other entities that promote I4.0. The third and final limitation concerns the still general nature of the risk mitigation plans presented. These plans lack greater detail and must be adjusted to the particularities and specific contexts of each company. Thus, it is proposed that future research include a new phase of interaction with stakeholders to detail and customize mitigation and capacity development measures, considering the particularities of each organizational structure.

From a theoretical perspective, this study contributes to the literature by integrating risk management into strategic scenario development within the context of I4.0. The proposed framework extends existing approaches by linking risk assessment with strategic positioning, offering a structured methodology that supports decision-making under uncertainty. From a practical and managerial perspective, the framework provides organizations with a tool to align their risk exposure with their risk appetite, enabling more informed strategic choices. In particular, the identification of risk categories and their positioning within different strategic scenarios supports managers in prioritizing investments, defining mitigation strategies, and enhancing organizational readiness for digital transformation.

From a policy perspective, the results highlight the importance of coordinated actions between public institutions and industry stakeholders. For example, the identification of high-impact risks related to workforce skills (e.g., R6) suggests that targeted public-private partnerships can play a key role in reducing critical vulnerabilities. Initiatives supported by European Union funding programs, such as Portugal 2030, can be leveraged to develop training programs, foster knowledge transfer, and support the upskilling of the workforce. These measures can contribute to reducing high-risk exposure and facilitating the transition toward more advanced strategic scenarios, such as “Capability” strategies.

## Data Availability

Due to institutional policy and data privacy issues, these datasets are not publicly available. However, interested parties may request access to them by contacting Vítor Alcácer at vitor.alcacer@estsetubal.ips.pt. No proprietary or third-party data was used in this study.

## Appendix

**Table A.1:** Articles of the first SLR

**Database**	**Article name**	**Methodology used to develop scenarios**	**Comments**	**References**
SCOPUS & WoS	Global Footprint Design based on genetic algorithms - An "Industry 4.0" perspective	Genetic algorithms analyze different production network scenarios, comparing network structures and costs over time.	The objective was to identify a path in the trade-off between the costs of migrating from one network structure to another and the total costs of the series of future network scenarios. The methodology allows decision-makers to develop improved strategies for developing their companies, identifying migration paths with minimized effort.	^59^
SCOPUS	Geography, Geographers and the Future Scenario: Malaysia	It does not explicitly detail a specific methodology but mentions that since the early 2000, four future scenarios have been generated to chart the development of higher education in Malaysia.	The objective of the study was to explore how geography as a discipline and geographers as practitioners of this discipline need to adapt to the demands of I4.0. The study concludes that although I4.0 introduces new technologies that would reduce the need for real (physical) mobility, the real human experience, which is geography’s main concern, still needs to be defended.	^60^
SCOPUS & WoS	How human factors affect operators’ task evolution in Logistics 4.0	The study uses empirical case studies to explore human factors in Logistics 4.0, although the exact methodology is not detailed.	The objective of this study was to understand how the introduction of technology in logistics significantly affects the work of operators, opening up new possibilities and challenges. The conclusions indicate that technology tends to replace logistics operators not only in physically dangerous tasks but also in cognitive tasks that are stressful and repetitive.	^61^
SCOPUS	Scenarios for the digitalisation of the construction industry	Scenario axis methodology to develop four plausible future scenarios for industry transformation enabled by digital technologies.	The objective of the study included exploring how digital technologies can help address key construction challenges, emphasizing the importance of thinking and planning for the future, including unexpected events, to realize the benefits of digital technologies.	^62^
SCOPUS & WoS	Potential Scenarios and Hazards in the Work of the Future: A Systematic Review of the Peer-Reviewed and Gray Literatures	The study carried out a systematic review of published information to identify and characterize scenarios and hazards in the future of work.	The objective of the study was to anticipate the dangers that workers will face in the future. The study focuses on identifying and characterizing scenarios and dangers in the future of work.	^63^
SCOPUS & WoS	Propulsion Monitoring System for Digitized Ship Management: Preliminary Results from a Case Study	Not applicable.	The objective of the study was to provide operating models for the digitalized management of ships, based on the structured collection and processing of information, offering maritime companies effective decision support systems to strengthen their value chain.	^64^
SCOPUS & WoS	Current Status and Future Trends in the Operation and Maintenance of Offshore Wind Turbines: A Review	Not applicable.	The objectives of the study were to evaluate the benefits and limitations of O&M strategies, including automation and digitalization, and discuss integration implications in the early stages of offshore renewable energy projects.	^65^
SCOPUS & WoS	Foresight Readiness Assessment for Saudi Organizations	The study carried out a literature review on forecasting studies around the world and then a survey questionnaire was designed, tested, and administered online to organizations in government, private, and academic institutions.	The main objective of the study was to assess the readiness of Saudi organizations to adopt and practice forecasting methods in their strategies to achieve the strategic objectives set out in the National Vision 2030.	^66^
SCOPUS & WoS	From flexible electronics to flexible photonics: A brief overview	Not applicable.	The study aimed to highlight advances and challenges in the different routes to manufacturing flexible photonic devices.	^67^
SCOPUS & WoS	Future of Raw Materials Logistics	Comprehensive literature analysis to create reliable scenarios for the development of raw materials logistics chains.	The objective of the study was to create scenarios for the development of raw materials logistics chains and derive action recommendations for the supply chain. The study results in the creation of three scenarios for development and provides recommendations for dealing with supply chain challenges.	^68^
SCOPUS	Socio-Technical Process Modules for Configuring Current and Future Logistics Processes	This study is based on literature research, expert interviews, and workshops, and logistics process modules for modeling automotive logistics are derived.	The objective of this study was to contribute to the analysis of the impacts of I4.0 on logistics. This study enables a structured depiction of processes in the dispositive and operative logistics, allowing a direct comparison of current and future processes.	^69^
SCOPUS & WoS	Persona’s Role in the Design of Future Technologies by Academics and Practitioners	Analysis of collected data from a conference co-design workshop for future scenarios in different platforms and environments.	The objective of the study was to explore empirical differences in the perspectives and use of people as a design tool between academics and industry professionals. The study’s findings show that academics and industry professionals have different perspectives and approaches toward the design process and the use of design tools.	^70^
SCOPUS & WoS	Product-service systems evolution in the era of Industry 4.0	Focus group as a methodology to develop future scenarios and identify the main trajectories that will shape a future scenario in which PSS (Product-Service Systems) and I4.0 merge.	The objectives of the study included understanding the directions companies are taking to offer new value to their customers in the context of I4.0. The findings highlight that the introduction of I4.0 technologies can facilitate the delivery of personalized service functionalities, enabling new customer needs and expectations.	^71^
SCOPUS & WoS	Torn between digitized future and context dependent past - How implementing 'Industry 4.0' production technologies could transform the German textile industry	Empirical mixed methods in three textile clusters including a stakeholder workshop.	The objectives of this study were to explore how historical roots and context conditions can influence I4.0 production technologies in the German textile industry, and draft future scenarios based on the empirical research. The findings include the expected benefits of I4.0 and the influence of historical roots.	^72^
SCOPUS & WoS	Advanced robotics and additive manufacturing of composites: towards a new era in Industry 4.0	Not applicable.	The study aimed to portray current and future scenarios related to the application of I4.0 concepts in the manufacture of polymer composites.	^73^
SCOPUS	Analyzing Industry 4.0 trends through the Technology Roadmapping Method	SLR to search events for a Technological Roadmapping Method	The objective of this study was to better understand the main issues about I4.0 and the consequences and developments in future scenarios for the industrial sector.	^74^
SCOPUS & WoS	Energy-Efficient beyond 5G Multiple Access Technique with Simultaneous Wireless Information and Power Transfer for the Factory of the Future	Not applicable.	The objective of the study was to improve energy efficiency, considering the trade-offs between increasing efficiency and reducing energy consumption.	^75^
SCOPUS	Future Scenarios and the Most Probable Future for Next Generation Manufacturing	Scenarios are created based on the dimensions of governance, organization, capabilities, interfaces, and resilience.	The objective of this study was to portray a likely future of manufacturing in 2030, enabled by connected data (digital shadows) shared in inter-organizational data spaces.	^76^
SCOPUS	How Digital Shadows, New Forms of Human-Machine Collaboration, and Data-Driven Business Models Are Driving the Future of Industry 4.0: A Delphi Study	Real-time Delphi analysis to derive reliable future scenarios that showcase the next generation of manufacturing systems.	The objective of this study was to understand how the concept of the Internet of Production affects manufacturing and how managers and workers must deal with new forms of collaboration between humans, robots, intelligent machines, and algorithms.	^77^
WoS	Sustainability-Oriented Innovation Foresight in International New Technology Based Firms	Real-time Delphi analysis to derive reliable future scenarios that showcase the next generation of manufacturing systems.	The objective of the study was to understand how international NTBFs (New Technologies Based Firms) face technology and how this impacts sustainability-oriented innovation. The research aims to plan future scenarios for international NTBFs based on sustainability-oriented innovations.	^78^
